# Recipient‐derived macrophages mediate acute cardiac allograft rejection via GSDMD‐induced pyroptosis mechanism

**DOI:** 10.1002/ctm2.70729

**Published:** 2026-07-05

**Authors:** Bixian Luo, Zelai Wu, Chengyu Hu, Anqi Ni, Jun He, Hongming Liu, Weixun Xie, Fuping Li, Weihua Gong

**Affiliations:** ^1^ Department of Gastrointestinal Surgery Fuzhou University Affiliated Provincial Hospital Fuzhou Fujian China; ^2^ Department of Surgery Second Affiliated Hospital of School of Medicine, Zhejiang University Hangzhou China; ^3^ Department of Nephrology Fuzhou University Affiliated Provincial Hospital Fuzhou Fujian China

**Keywords:** GSDMD, heart transplantation, macrophages, pyroptosis

## Abstract

**Objective:**

To determine whether macrophage gasdermin D (GSDMD)‐dependent pyroptosis drives acute cardiac allograft rejection and whether targeting GSDMD improves graft survival.

**Methods:**

GSDMD activation was assessed by immunoblotting. Graft survival wasanalysed in global and macrophage‐specific GSDMD‐deficient recipients. Single‐cell RNA‐seq identified GSDMD‐expressing populations, while immune infiltration and cytokines were measured by flow cytometry and immunohistochemistry; IL1R1 knockout and cytokine challenge were used for validation.

**Results:**

GSDMD deficiency markedly prolonged graft survival. Single‐cell profiling showed increased GSDMD expression and pyroptosis primarily in recipient‐derived M1 macrophages during acute rejection. Macrophage‐specific GSDMD deficiency reduced CD8^+^T cell/macrophage infiltration and decreased TNF‐α and IL‐1β. TNFα/IL6 activated NF‐κB and JAK–STAT3 to upregulate GSDMD, and IL‐1β was verified as a key effector for CD8^+^T cells mediated‐rejection. GSDMD pyroptosis inhibitors mitigated rejection and extended graft survival.

**Conclusion:**

Recipient M1 macrophages promote acute rejection via TNFα/IL6‐driven NF‐κB/STAT3 activation of GSDMD‐mediated pyroptosis. Targeting GSDMD represents a promising strategy to enhance cardiac allograft survival.

## Graphical abstract

- Recipient‐derived macrophages undergo M1‐like polarization after heart transplantation under interferon, IL‐6 and TNF‐α signalling.
- NF‐κB/STAT3 activation promotes GSDMD‐mediated pyroptosis and IL‐1β release in macrophages.
- Macrophage‐derived IL‐1β enhances CD8^+^T cell recruitment and activation, driving acute cardiac allograft rejection.

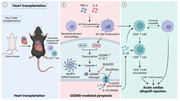

## 1 INTRODUCTION

Cardiac transplantation has become the standard treatment for end‐stage heart failure; however, ischemia–reperfusion (I/R) injury and acute rejection are inevitable complications that threaten graft survival.^1^ The extent of immune cell infiltration correlates with the severity of immune rejection during the acute phase.^2^ After transplantation, factors such as IRI, surgical trauma and donor injury lead to various forms of cell death in the graft, accompanied by the release of inflammatory cytokines. However, it remains unclear whether pyroptosis occurs in cardiac grafts and whether targeting pyroptosis can modulate inflammation, thereby affecting immune rejection and graft survival.

Pyroptosis is a form of programmed cell death mediated by the gasdermin (GSDM) family of proteins.^3^ This process is characterized by the cleavage of gasdermin by caspases, which causes the N‐terminal fragment to form pores in the cell membrane, leading to cell swelling, membrane rupture, and the release of inflammatory mediators, such as IL‐1β and IL‐18.^4^ Among the gasdermins, GSDMD is considered the classical mediator of pyroptosis, and the canonical pyroptosis pathway follows the sequence NLRP3‐CASPASE1‐GSDMD‐IL1β.^5^, ^6^ In the heart, NLRP3 inhibitors have been shown to alleviate IRI‐induced injury.^7^ In mouse heart transplantation model, treatment with caspase‐1 inhibitor significantly suppressed acute rejection of the graft and extended graft survival time.^8^ Recent studies reported GSDMD as a new target for improved ventricular remodelling and reduced heart failure after acute myocardial infarction.^9^ Other study proved that mutations in GSDMD confer protection against renal I/R injury.^10^ Previous studies have demonstrated that inflammatory cytokines, including TNF‐α, IL‐6, and IL‐1β, are upregulated in heart grafts after transplantation, indicating an increase in the inflammatory response.^11^, ^12^, ^13^ Recent studies have shown that during the early stages of acute organ rejection in liver transplantation, inflammasomes are responsible for initiating the immune response, leading to the upregulation of IL‐1β.^14^ Similarly, recent reports have indicated an increase in IL‐1β expression levels in heart allografts.^15^ However, the exact role of pyroptosis in this process, including the specific cell types involved and the mechanisms underlying its activation, remains poorly understood. This study seeks to investigate the occurrence of pyroptosis in heart grafts during the acute rejection phase and explore its role in immune rejection.

Macrophages play a central role in the inflammatory response, with M1 macrophages promoting inflammation through the secretion of cytokines such as TNF‐α, IL‐1β and iNOS.^16^, ^17^ During the acute phase of transplantation, M1 macrophages are particularly important, as they significantly contribute to immune rejection by activating endothelial cells and promoting T cell responses.^18^, ^19^ Recent studies have shown that after liver transplantation, M1 macrophages secrete inflammatory mediators that affect the graft's immune microenvironment, contributing to graft damage.^20^ Reviews have highlighted that, following liver transplantation, macrophages release pro‐inflammatory cytokines such as IL‐1, IL‐18, TNF‐α and IFN‐γ, which play roles in both immune rejection and tolerance.^21^ During acute rejection in kidney transplantation, renal macrophages adopt an M1 pro‐inflammatory phenotype and secrete various cytokines, including IFN‐γ, IL‐1β, IL‐18 and TNF‐α, which activate endothelial cells and promote cytotoxic T cell production.^22^, ^23^ Additionally, activated inflammatory macrophages generate ROS and RNS, further intensifying allograft injury.^24^ In heart transplantation, pro‐inflammatory factors like TNF‐α and IL‐6 are upregulated during acute rejection,^25^ and both the NF‐κB and JAK/STAT3 signalling pathways play key roles in mediating T cell activation and cytokine production, driving the inflammatory response.^26^, ^27^, ^28^ It is critical to understand whether these signalling pathways in macrophages contribute to the upregulation of GSDMD‐mediated pyroptosis and how they influence immune rejection.

The aim of this study is to investigate the occurrence of pyroptosis in heart grafts during acute rejection, identify the specific cell types involved, and elucidate the molecular mechanisms driving the upregulation of GSDMD‐mediated pyroptosis. Additionally, we will explore the role of key inflammatory factors in promoting immune rejection, focussing on IL‐1β as potential mediators. Ultimately, this research will provide a better understanding of the role of pyroptosis in heart transplantation and offer insights into potential therapeutic strategies for targeting GSDMD‐mediated pyroptosis to reduce immune rejection, decrease inflammatory responses, and improve graft survival.

## 2 METHODS

### 2.1 Mice obtained

Male C57BL/6 and BALB/c mice (8–12 weeks old) were obtained from Shanghai SLAC Laboratory Animal Co., Ltd. The GSDMD knockout (GSDMD^−/Ȓ^) mice, IL1R1 knockout (IL‐1R1^−/−^) mice, GSDMD^floxp/floxp^ (GSDMD‐WT), and Lyz2^cre^‐GSDMD^floxp/floxp^ (GSDMD‐CKO) mice were sourced from Institute of Immunology, Zhejiang University School of Medicine. All mice were housed in a specific pathogen‐free (SPF) environment with controlled temperature, humidity, and a 12‐h light/dark cycle, with ad libitum access to food and water. The study protocol was approved by the Zhejiang University Animal Center and conducted under the approved protocol license (IACUC‐ZJU20220256).

#### 2.1.1 Mouse genotyping

Mouse genotypes were verified by PCR using genomic DNA prepared from tail biopsies. Briefly, 2–3 mm tail tips were collected from mice at weaning and lysed in NaOH solution by heating. The lysates were then neutralized with Tris‐HCl buffer and centrifuged briefly, and the supernatants were used as PCR templates. Genotype‐specific PCR was performed to identify the wild‐type, floxed, knockout and Cre alleles of the indicated mouse strains, including GSDMD^−/−^ mice, GSDMD‐WT mice, GSDMD‐CKO mice, and Il1r1^−/−^ mice. PCR products were separated by agarose gel electrophoresis and visualized under ultraviolet illumination. Genotypes were determined according to the expected allele‐specific band patterns.

### 2.2 Mice cardiac transplantation

After anaesthesia, heterotopic cervical cardiac transplantation was performed using a cuff technique. The external jugular vein and common carotid artery of recipient mice were exposed and everted over pre‐prepared cuffs. Donor hearts were harvested with the pulmonary artery and ascending aorta preserved, while the remaining vessels were ligated. The donor pulmonary artery was anastomosed end‐to‐end to the recipient external jugular vein, and the donor ascending aorta was connected to the recipient common carotid artery. Graft function was monitored daily by palpation, and graft loss was defined as complete cessation of palpable heartbeat. Heart grafts were collected on postoperative Days 1, 3 and 5 for western blotting, RT‐qPCR, H&E staining, immunohistochemistry, immunofluorescence and flow cytometry analyses. For rescue experiments, recombinant mouse IL‐1 (MCE, HY‐P7073; 100 g/kg) or vehicle was administered intraperitoneally every other day after transplantation until graft harvest or graft loss. For pharmacological inhibition, recipients received NU6300 (MCE, HY‐18930; 10 mg/kg); disulfiram (MCE, HY‐B0240; 50 mg/kg); the NF‐B inhibitor BAY 11–7082 (MCE, HY‐13453; 10 mg/kg); the STAT3 inhibitor Stattic (MCE, HY‐13818; 10 mg/kg); or vehicle intraperitoneally every other day after transplantation until graft harvest or graft loss.

### 2.3 Cell culture

RAW264.7 macrophages and HEK293T cells were cultured in DMEM supplemented with 10% foetal bovine serum and 1% penicillin‐streptomycin at 37°C in 5% CO. RAW264.7 cells were stimulated with recombinant mouse TNF‐α (MCE, HY‐P7090) at 20 or 40 ng/mL, or recombinant mouse IL‐6 (MCE, HY‐P7063) at 20 or 40 ng/mL, for 24 h. For subsequent mechanistic assays, TNF‐ and IL‐6 were used at 40 ng/mL each unless otherwise indicated. For inhibitor dose‐response experiments, cells were pretreated with NU6300 (MCE, HY‐18930) or disulfiram (MCE, HY‐B0240) for 1 h before cytokine stimulation; the inhibitor concentrations are indicated in the corresponding figure legends.

### 2.4 Western blot

Heart grafts and cultured cells were lysed in RIPA buffer supplemented with protease and phosphatase inhibitors. Tissue samples were homogenized using a tissue grinder and centrifuged at 12 000 rpm for 10 min at 4°C. Protein concentrations were determined using an Enhanced BCA Protein Assay Kit (Beyotime). Equal amounts of protein were separated by SDS‐PAGE and transferred onto PVDF membranes. After blocking with 5% non‐fat milk, membranes were incubated with primary antibodies overnight at 4°C, followed by incubation with HRP‐conjugated secondary antibodies at room temperature. Protein bands were visualized using an enhanced chemiluminescence detection system and quantified using ImageJ software. The primary antibodies used are listed in Table S1.

### 2.5 Flow cytometry

Cardiac grafts were weighed before digestion, minced and enzymatically digested with 1 mg/mL protease and 0.8 mg/mL collagenase IV for 30 min at 37°C. Single‐cell suspensions were filtered through a 70 m strainer, and erythrocytes were lysed with red blood cell lysis buffer. Spleens were mechanically dissociated and processed in the same manner. For intracellular cytokine staining, single‐cell suspensions were stimulated with a cell stimulation cocktail in the presence of protein transport inhibitors for 4 h at 37°C. Cells were then stained with viability dye and surface antibodies for 30 min at 4°C, followed by fixation/permeabilization and intracellular staining for TNF‐α, IL‐6, IL‐1β. Gating was performed on live single cells, followed by CD45^+^ leukocytes. T cells were defined as CD45^+^CD3^+^ cells, CD8^+^‐T cells as CD45^+^CD3^+^CD8^+^ cells, and macrophages as CD45^+^CD11b^+^F4/80^+^ cells. M1‐like and M2‐like macrophages were evaluated using CD86 and CD206, respectively. The estimated absolute number of target cells per graft was calculated as: total viable cells recovered from the graft × percentage of target cells among live single cells. Target cells per mg graft tissue were calculated as: estimated absolute number of target cells per graft / graft weight (mg). Data were acquired on a BD LSRFortessa X‐20 and analysed using FlowJo. Antibody details are provided in Table S2.

### 2.6 Quantitative polymerase chain reaction (qPCR)

Total RNA was isolated from mouse heart grafts or cultured cells using the mRNA Isolation Kit (R711‐02, Vazyme, China) according to the manufacturer's instructions. RNA samples were reverse‐transcribed into cDNA with the HiScript II Reverse Transcriptase Kit (R223‐01, Vazyme). Quantitative real‐time PCR was performed using ChamQ Universal SYBR qPCR Master Mix (Q711‐02, Vazyme) on a CFX96 Opus Real‐Time PCR System (Bio‐Rad). Relative gene expression was calculated using the 2−Ct method. Primer sequences are listed in Table S3.

### 2.7 Immunohistochemistry (IHC) analysis

Paraffin‐embedded mouse cardiac graft sections were deparaffinized in xylene and rehydrated through a graded ethanol series. After antigen retrieval, endogenous peroxidase activity was blocked, and sections were incubated with blocking buffer to reduce nonspecific staining. The sections were then incubated with primary antibodies overnight at 4°C, followed by HRP‐conjugated secondary antibodies at room temperature. Immunoreactivity was visualized using DAB substrate, and nuclei were counterstained with haematoxylin. Images were acquired using a light microscope. For quantification, the number of positively stained cells was counted in five randomly selected fields from each section using Image‐Pro Plus 6.0. The antibodies used are listed in Table S4.

### 2.8 Immunofluorescent staining

Heart grafts were collected and fixed overnight at 4°C in 4% paraformaldehyde. After fixation, tissues were washed with PBS, cryoprotected in 30% sucrose overnight at 4°C, embedded in OCT compound, and sectioned into 8–10 m slices. Sections were blocked with 5% goat serum containing 0.1% Triton X‐100 for 1 h at room temperature and then incubated with primary antibodies overnight at 4°C. After washing with PBS, sections were incubated with fluorescent secondary antibodies for 2 h at room temperature. Nuclei were counterstained with DAPI, and images were acquired using a Zeiss LSM 800 confocal microscope. For quantification, the number of positively stained cells or double‐positive cells was counted in five randomly selected fields from each section using Image‐Pro Plus 6.0. The antibodies used are listed in Table S4.

### 2.9 Dual‐luciferase reporter assay

HEK293T cells were seeded in 24‐well plates and co‐transfected with a mouse Gsdmd promoter firefly luciferase reporter plasmid, NF‐B or STAT3 expression plasmids, and a Renilla luciferase plasmid as an internal control. Empty vector was used as the negative control. All plasmids were purchased from Miaoling Biotechnology Co., Ltd. At 48 h after transfection, firefly and Renilla luciferase activities were measured using the Dual‐Luciferase Reporter Assay System (Vazyme, DL101) according to the manufacturer's instructions. Relative promoter activity was calculated by normalizing firefly luciferase activity to Renilla luciferase activity.

### 2.10 Cell sorting

Cardiac grafts and recipient spleens were harvested on postoperative day 5 and processed into single‐cell suspensions as described above. CD68^+^ macrophage‐enriched cells were isolated from graft and splenic single‐cell suspensions by flow cytometry‐based cell sorting using a Beckman Coulter MoFlo Astrios EQ cell sorter. Sorted CD68^+^ cell fractions from cardiac grafts and recipient spleens were collected separately and used for downstream qPCR, western blotting and functional analyses.

### 2.11 Single‐cell RNA library construction, sequencing and data processing

Single‐cell RNA‐seq data of mouse cardiac allografts undergoing acute rejection were obtained from our previously published study. The raw sequencing data are publicly available in the Genome Sequence Archive under accession number CRA005239. In the original study, non‐cardiomyocytes were isolated from mouse cardiac grafts harvested on postoperative Days 0, 1, 3 and 5 and subjected to single‐cell RNA sequencing using the Chromium Single Cell 5′ Library and Gel Bead Kit according to the manufacturer's protocol. Raw sequencing reads were processed using Cell Ranger software (v6.0.1, 10x Genomics) with the mouse reference genome mm10 to generate gene–cell unique molecular identifier matrices.

For the present study, the published scRNA‐seq dataset was reanalysed to investigate macrophage dynamics and GSDMD‐related inflammatory signatures during acute cardiac allograft rejection. Potential doublets were identified using DoubletDetection, Scrublet and DoubletFinder, and cells predicted as doublets by at least two of these tools were excluded. Low‐quality cells were further removed according to the following criteria: cells with fewer than 1000 or more than 15 000 UMI counts, fewer than 500 or more than 3000 detected genes, or more than 5% mitochondrial gene‐derived UMIs. After quality control, 45 959 high‐quality cells were retained for downstream analysis. Donor/recipient origin was assigned to each cell using the souporcell algorithm. 

Downstream analyses were performed using Seurat. UMI counts were normalized using the LogNormalize method, followed by identification of highly variable genes, data scaling, principal component analysis, clustering and UMAP visualization. Cell types were annotated according to canonical lineage‐specific marker genes. Macrophages were identified based on representative macrophage‐associated markers, and macrophage subsets were further classified according to established M1‐like, M2‐like and resident macrophage‐related gene signatures. The representative marker genes used for macrophage subset annotation are shown in the corresponding figure legends and Supporting Information (Figures). Differential gene expression and pathway enrichment analyses were performed to evaluate GSDMD‐associated inflammatory changes in macrophage subsets during rejection progression.

### 2.12 Macrophage‐CD8^+^T cell transwell co‐culture Assay

BALB/c donor hearts were transplanted into GSDMD‐WT or GSDMD‐CKO recipient mice on a C57BL/6 background. On postoperative Day 5, cardiac grafts and recipient spleens were harvested, and CD68^+^ macrophage‐enriched cells were isolated by flow cytometry‐based sorting. CD8^+^T cells were isolated from WT C57BL/6 spleens and activated with anti‐CD3/CD28 before co‐culture.

Activated CD8^+^T cells were seeded in the upper chamber at 1 × 10^5^ cells per well. The lower chamber contained control medium, GSDMD‐WT Day 5 macrophages, GSDMD‐CKO Day 5 macrophages, GSDMD‐WT Day 5 macrophages plus anti‐IL‐1β neutralizing antibody, or GSDMD‐CKO Day 5 macrophages plus recombinant IL‐1β. For macrophage‐containing groups, 1 × 10^5^ sorted CD68^+^ macrophage‐enriched cells were added to the lower chamber and stimulated with TNF‐α and IL‐6. After 24 h of co‐culture, migrated CD8^+^T cells and cytokine‐producing CD8^+^T cells were analysed by flow cytometry.

### 2.13 Statistical analysis

Statistical analyses were performed using GraphPad Prism 9.0. Data are presented as mean ± SEM. The number of biologically independent samples is indicated in each figure legend. Survival curves were analysed using the log‐rank test. Two‐group comparisons were performed using two‐tailed Student's *t*‐test. Multiple‐group comparisons were performed using one‐way ANOVA. *p* < .05 was considered statistically significant.

## 3 RESULTS

### 3.1 GSDMD‐mediated pyroptosis was upregulated during the acute immune rejection stage of mouse cardiac transplantation

**FIGURE 1:**
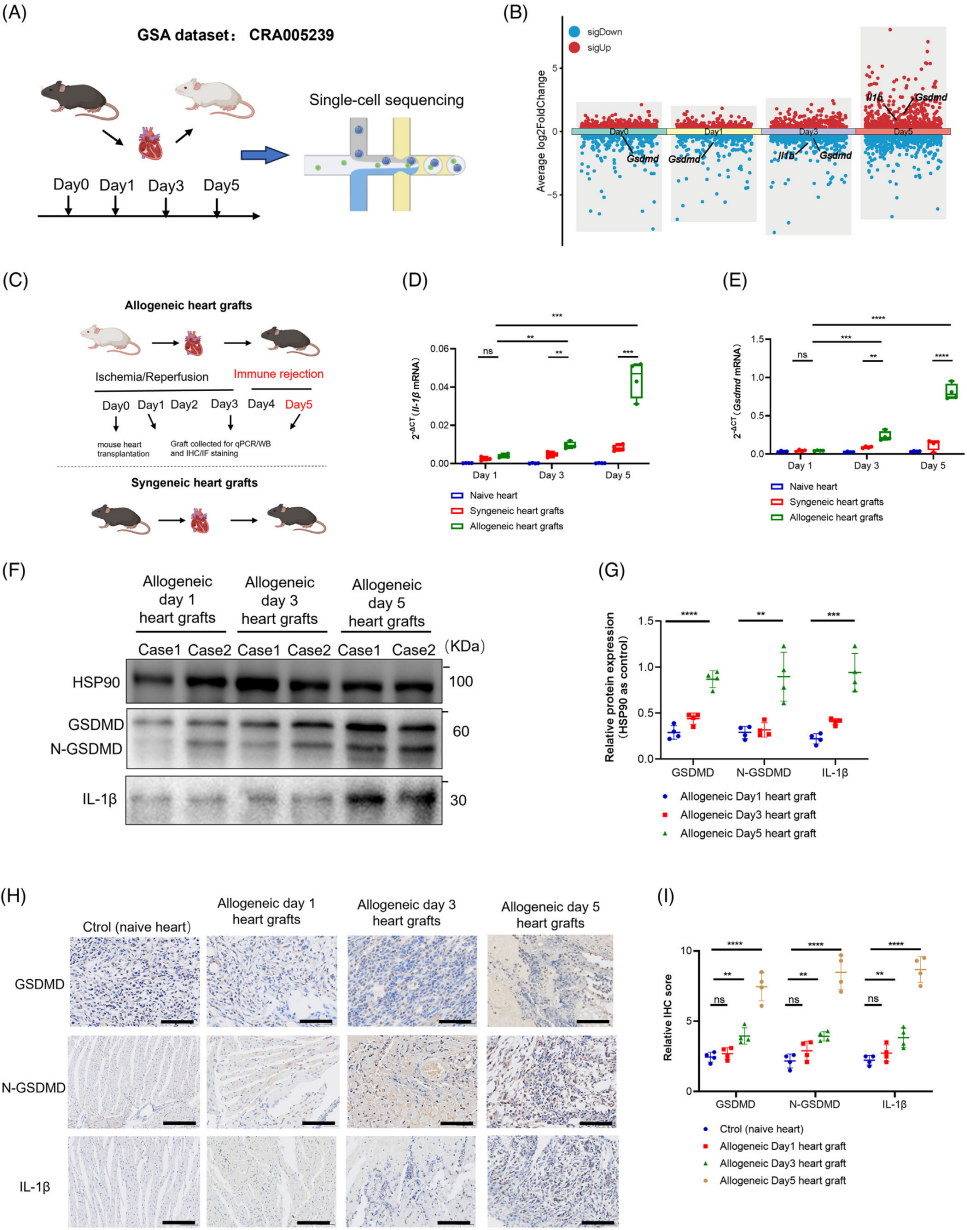
GSDMD‐mediated pyroptosis was upregulated during the acute immune rejection stage of mouse cardiac transplantation. (A) Schematic showing reanalysis of the previously published scRNA‐seq dataset of mouse cardiac grafts collected at Day 0, Day 1, Day 3 and Day 5 post‐transplantation (GSA dataset: CRA005239). (B) Differential expression analysis showing changes in pyroptosis‐related genes, including Gsdmd and Il1b, during rejection progression. (C) Schematic illustration of sampling time points. Days 1–3 was considered the late ischemia/reperfusion stage, whereas Day 5 was defined as the acute immune rejection stage. (D and E) qPCR analysis of Il1b and Gsdmd mRNA expression in naïve hearts, syngeneic grafts, and allogeneic grafts. (F and G) Representative western blot images and quantification of GSDMD, N‐GSDMD, and IL‐1β expression in allogeneic grafts. (H and I) Representative immunohistochemical staining and quantification of GSDMD, N‐GSDMD and IL‐1β in naïve hearts and allogeneic grafts. For D, E, G and I, *n* = 4 biologically independent samples per group. Data are presented as mean ± SEM. ns, not significant; **p < .01; ***p < .001; ****p < .0001.

To investigate the temporal dynamics of GSDMD‐mediated pyroptosis during cardiac transplantation, we reanalysed our team previously published single‐cell RNA sequencing dataset of mouse heart grafts collected at Day 0, Day 1, Day 3 and Day 5 post‐transplantation (GSA dataset: CRA005239) (Figure 1A). This analysis revealed increased expression of Gsdmd and Il1b in heart grafts, particularly at Day 5 (Figure 1B). Based on the progression of the mouse cardiac transplantation model, Days 1–3 was considered the late ischemia/reperfusion stage, whereas Day 5 represented the acute immune rejection stage (Figure 1C). qPCR analysis showed that Il1b and Gsdmd mRNA levels were mildly increased during Days 1–3 and markedly upregulated at Day 5 in allogeneic heart grafts (Figure 1D,E). Consistently, western blot analysis demonstrated a pronounced increase in GSDMD, N‐GSDMD and IL‐1 protein expression in Day 5 allogeneic heart grafts (Figure 1F,G). Immunohistochemical staining further corroborated these findings, showing stronger expression of GSDMD, N‐GSDMD and IL‐1 in grafts during the acute immune rejection stage (Figure 1H,I). These results suggest that GSDMD‐mediated pyroptosis is enhanced in heart grafts and is most prominent during acute immune rejection.

### 3.2 GSDMD deficiency reduced immune infiltration and prolonged cardiac allograft survival

**FIGURE 2:**
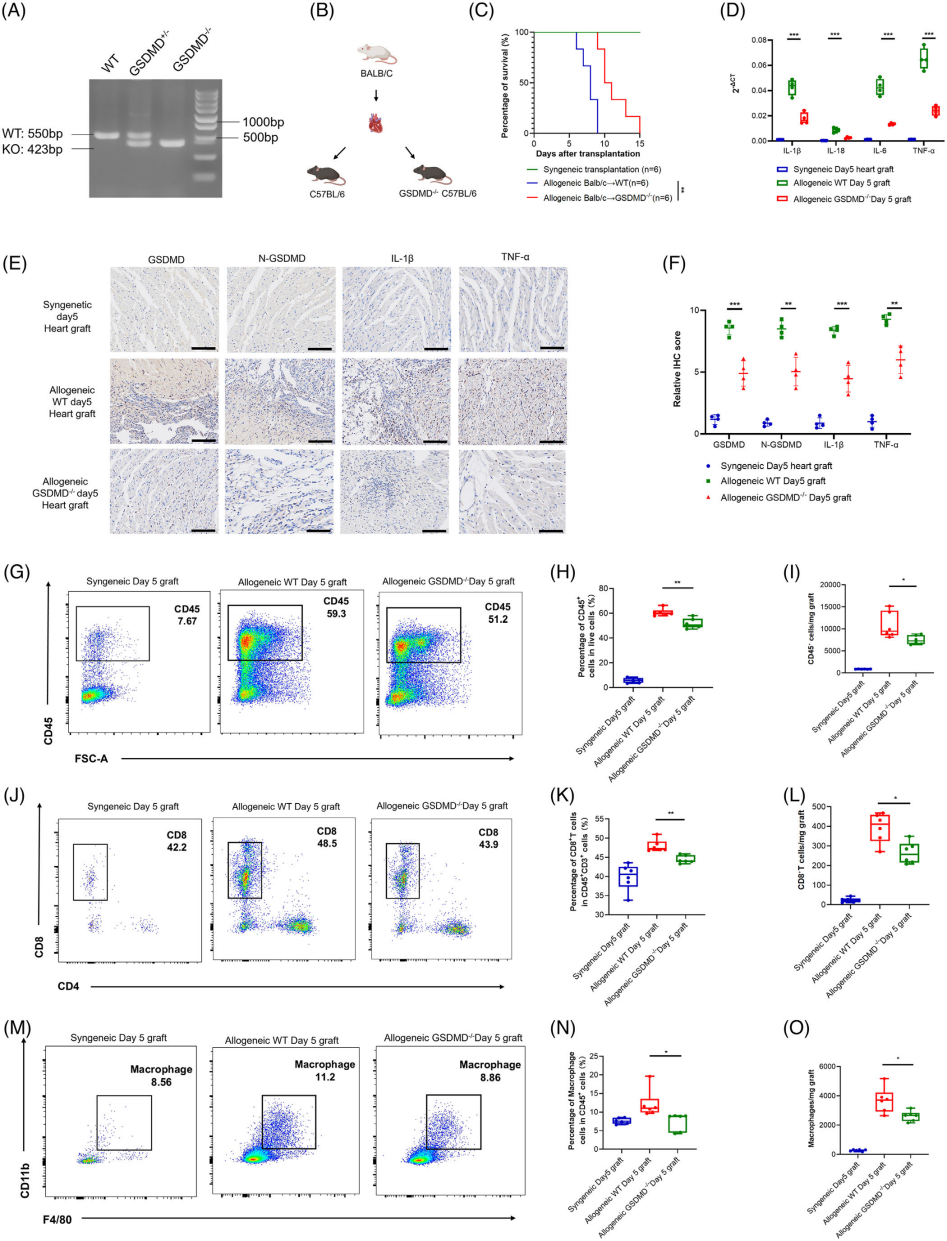
Allografts transplanted into GSDMD^−/−^ mice reduced pyroptosis and have prolonged survival. (A) PCR‐based genotyping of WT, GSDMD^+/−^ , and GSDMD^−/−^ mice. The WT allele was detected at 550 bp, and the knockout allele was detected at 423 bp. (B) Schematic illustration of BALB/c donor hearts transplanted into WT or Gsdmd−/− recipients on a C57BL/6 background. (C) Kaplan–Meier survival curves of cardiac grafts. *n* = 6 mice per group. (D) qPCR analysis of Il1b, Il18, Il6 and Tnfα mRNA expression in Day 5 grafts. *n *= 4 biologically independent samples per group. (E and F) Representative immunohistochemical staining and quantification of GSDMD, N‐GSDMD, IL‐1β and TNF‐α in Day 5 grafts. *n* = 4 biologically independent samples per group. (G–I) Representative flow cytometry plots and quantification of infiltrating CD45^+^ leukocytes in Day 5 grafts. *n* = 5 biologically independent samples per group. (J–L) Representative flow cytometry plots and quantification of infiltrating CD8^+^T cells in Day 5 grafts. *n* = 5 biologically independent samples per group. (M–O) Representative flow cytometry plots and quantification of infiltrating macrophages in Day 5 grafts. *n* = 5 biologically independent samples per group. Data are presented as mean ± SEM. ns, not significant; *p < .05; **p < .01; ***p < .001.

To assess the impact of GSDMD on cardiac transplant survival and immune rejection responses, we performed transplantation experiments using WT and GSDMD^−/−^ mice. Genotyping confirmed successful knockout of GSDMD in mice (Figure 2A). BALB/c donor hearts were transplanted into WT or GSDMD^−/−^ recipients on a C57BL/6 background, with syngeneic transplantation as controls (Figure 2B). Compared with WT recipients, GSDMD^−/−^ recipients showed prolonged cardiac allograft survival (Figure 2C). H&E staining of cardiac grafts is shown in Figure S1A. qPCR analysis revealed that the mRNA levels of inflammatory markers, including Il1b, Il18, Il6 and Tnfα, were reduced in Day 5 allografts from GSDMD^−/−^ recipients compared with WT recipients (Figure 2D). Immunohistochemical staining further showed decreased GSDMD, N‐GSDMD, IL‐1β and TNF‐α expression in allografts from GSDMD^−/−^ recipients (Figure 2E,F), indicating reduced inflammatory response and pyroptosis.

Flow cytometry analysis showed that Day 5 allogeneic grafts from GSDMD^−/−^ recipients exhibited reduced immune‐cell infiltration compared with WT recipients. The percentages and absolute numbers of infiltrating CD45^+^ leucocytes, CD8^+^T cells, and macrophages were all decreased in allografts from GSDMD^−/−^ recipients (Figure 2G–O). These results indicate that GSDMD deficiency attenuates both innate macrophage infiltration and adaptive CD8^+^T cell responses during acute cardiac allograft rejection. We further transplanted WT or GSDMD^−/−^ donor hearts into WT recipients and found that GSDMD^−/−^ donor hearts survived longer than WT donor hearts (Figure S1B,C). Immunofluorescence staining further showed increased GSDMD expression both in CD68^+^ macrophages and cardiomyocytes within Day 5 allogeneic WT grafts (Figure S1D). Together, these findings suggest that GSDMD deficiency attenuates pyroptosis‐associated inflammation and immune‐cell infiltration during acute cardiac allograft rejection.

### 3.3 Increased M1 macrophage expression and its GSDMD‐mediated pyroptosis during acute cardiac rejection

To investigate macrophage phenotypic changes during cardiac transplantation, we analysed the single‐cell RNA sequencing dataset of cardiac grafts collected on Days 0, 1, 3 and 5 post‐transplantation. Cell populations were annotated according to canonical marker genes, and the expression of representative markers was shown by dot plot analysis (Figure S2A). UMAP visualization identified the major cell populations in cardiac grafts and showed their distribution across different time points (Figure S2B,C). Quantitative analysis of cell composition showed that the proportion of macrophages markedly increased at Day 5 and became the dominant cell population during the acute rejection stage, suggesting that macrophages may play an important role in acute cardiac allograft rejection (Figure S2D).

**FIGURE 3:**
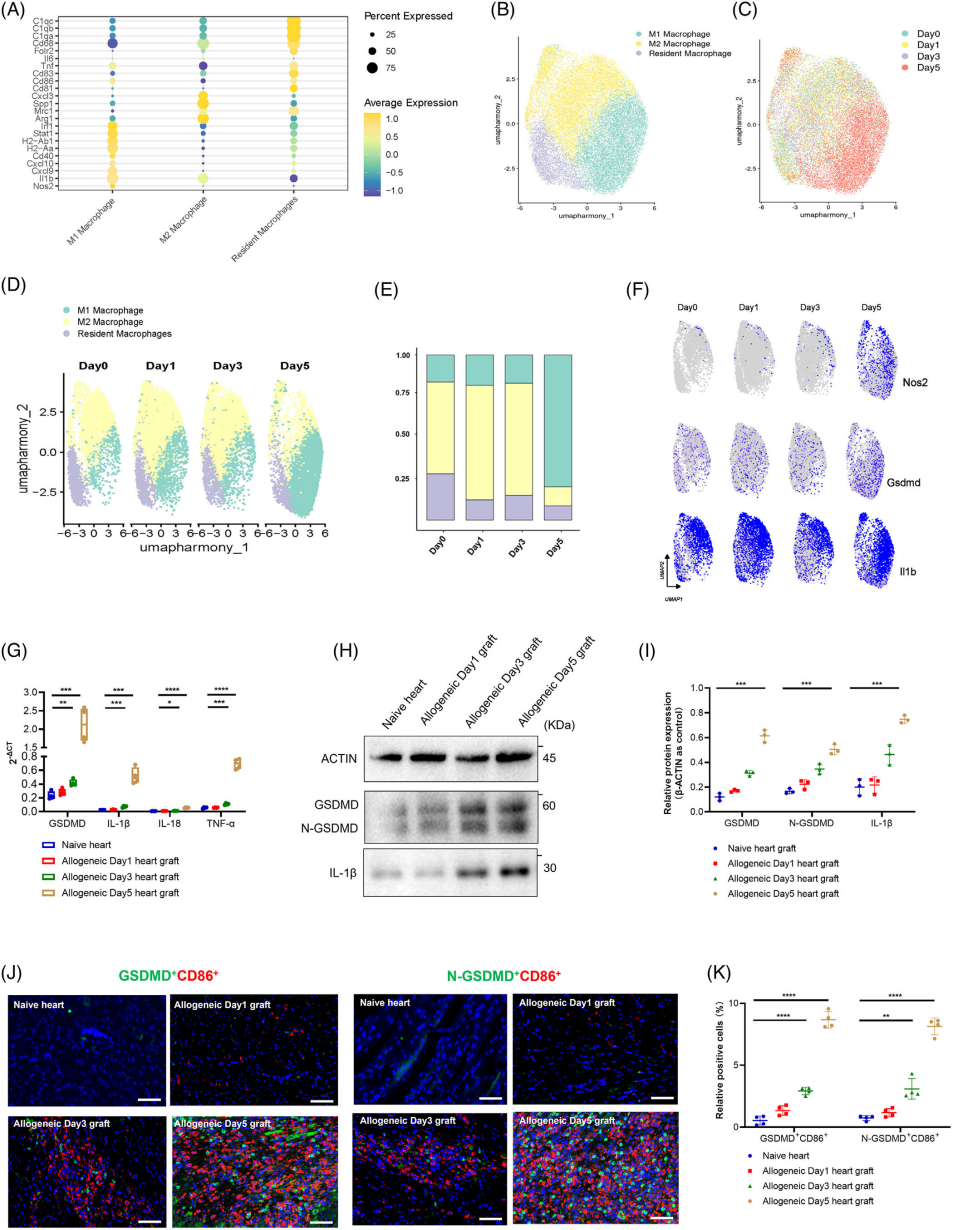
Increased M1 macrophage expression and its GSDMD‐mediated pyroptosis during acute cardiac rejection. (A) Dot plot showing the average expression levels of representative marker genes used for macrophage subset annotation, including M1‐like macrophages (Nos2, Il1b, Cxcl9, Cxcl10, Cd40, H2‐Aa, H2‐Ab1, Stat1 and Irf1), M2‐like macrophages (Arg1, Mrc1, Spp1 and Cxcl3), and resident macrophages (Folr2, C1qa, C1qb and C1qc). Cd68 was used as a macrophage‐associated marker to support macrophage identity. (B) UMAP visualization of M1‐like, M2‐like, and resident macrophage populations in cardiac grafts. (C) UMAP visualization of macrophages coloured by sampling time points. (D) UMAP plots showing macrophage subset distribution at Day 0, Day 1, Day 3 and Day 5 post‐transplantation. (E) Proportional distribution of macrophage subsets across different time points. (F) Feature plots showing Nos2, Gsdmd and Il1b expression in macrophages at the indicated time points. (G) qPCR analysis of Gsdmd, Il1b, Il18 and Tnfα mRNA expression in sorted macrophage‐enriched cells from nave hearts and allogeneic grafts. *n* = 4 biologically independent samples per group. (H and I) Representative western blot images and quantification of GSDMD, N‐GSDMD and IL‐1β expression in sorted macrophage‐enriched cells. *n* = 3 biologically independent samples per group. (J and K) Representative immunofluorescence staining and quantification of GSDMD^+^CD86^+^ and N‐GSDMD^+^CD86^+^ cells in grafts. *n* = 4 biologically independent samples per group. Data are presented as mean ± SEM. *p < .05; **p < .01; ***p < .001; ****p < .0001.

We next focussed on macrophage subsets. Dot plot analysis of representative markers supported the annotation of M1‐like, M2‐like, and resident macrophage populations (Figure 3A). Feature plots of the macrophage marker genes Cd68 and Lyz2 further supported the identification of macrophage populations in the grafts (Figure S2E). UMAP visualization showed the distribution of macrophage subsets in cardiac grafts and across different time points (Figure 3B–D). Proportional analysis revealed a marked increase in M1‐like macrophages at Day 5, accompanied by a relative decrease in M2‐like macrophages as rejection progressed (Figure 3E). Compared with the Days 1–3 ischemia/reperfusion stage, M1‐like macrophages sharply increased during the Day 5 acute rejection stage and accounted for more than 75% of total macrophages (Figure 3E). Feature plots further showed that the M1‐like inflammatory gene Nos2 was markedly increased at Day 5, together with enriched Gsdmd and Il1b expression in macrophages, suggesting enhanced GSDMD‐associated pyroptosis and pyroptosis‐related inflammatory signalling during acute rejection (Figure 3F).

We further compared Gsdmd with another gasdermin family member, Gsdme. Gsdme showed relatively low expression in non‐cardiomyocytes and limited expression in macrophages, whereas Gsdmd was enriched in macrophage regions (Figure S3A,B). Among major graft cell populations, macrophages exhibited the highest average Gsdmd expression (Figure S3C). Further comparison among macrophage subsets showed that Gsdmd expression was higher in M1‐like macrophages than in M2‐like and resident macrophage populations (Figure S3D). Additional subclustering revealed further heterogeneity within M1‐like and M2‐like macrophage populations (Figure S4). M1‐like macrophages could be further classified into antigen‐presenting M1‐like macrophages, inflammatory M1‐like macrophages, monocyte‐derived inflammatory macrophages, and IFN‐responsive M1‐like macrophages (Figure S4A). M2‐like macrophages were further divided into Trem2‐associated lipid‐associated macrophages, M2a macrophages, M2c macrophages and reparative macrophages (Figure S4B). These results further support the dynamic remodelling of macrophage phenotypes during acute cardiac allograft rejection.

To validate the single‐cell findings at the transcript and protein levels, we sorted macrophage‐enriched cells from grafts and spleens after transplantation. Consistent with the single‐cell analysis, qPCR showed that the mRNA levels of Gsdmd, Il1b, Il18 and Tnfα were increased in macrophages from allogeneic grafts, especially at the Day 5 acute rejection stage (Figure 3G). Western blot analysis further confirmed increased GSDMD, N‐GSDMD and IL‐1β protein expression in macrophages during rejection progression (Figure 3H,I). Immunofluorescence staining showed increased co‐localization of GSDMD or N‐GSDMD with CD86^+^ （M1‐like marker）cells in Day 5 allogeneic grafts (Figure 3J). Quantification further confirmed an increase in GSDMD^+^CD86^+^ and N‐GSDMD^+^CD86^+^ cells at Day 5 (Figure 3K). Together, these findings indicate that M1‐like macrophage accumulation accompanied by enhanced GSDMD‐associated pyroptosis during acute cardiac allograft rejection.

### 3.4 Majority of macrophages are derived from the transplant recipient during acute cardiac rejection

**FIGURE 4:**
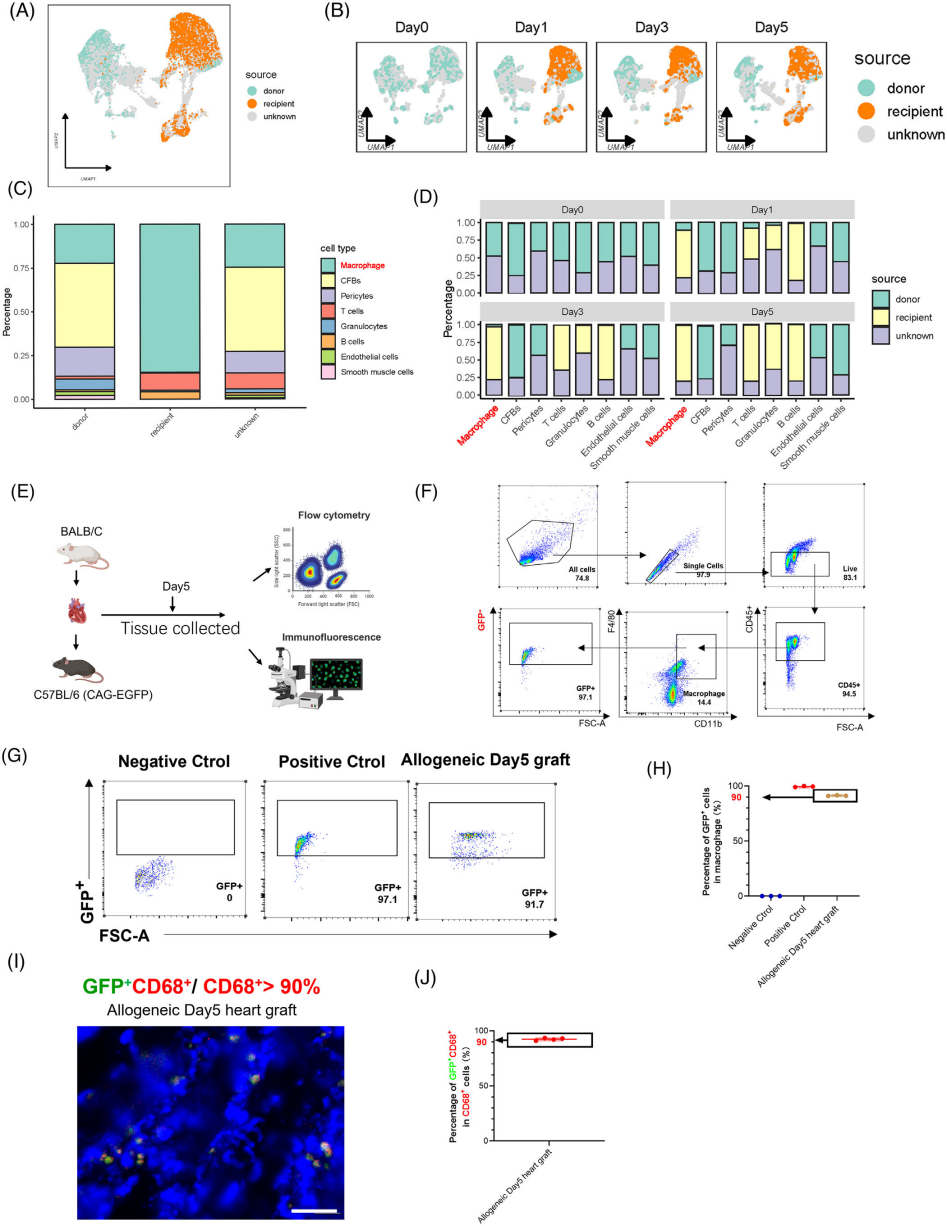
The majority of macrophages are derived from the transplant recipient during acute cardiac rejection. (A) UMAP visualization showing donor‐derived, recipient‐derived, and unknown‐origin cells in cardiac grafts. (B) UMAP plots showing donor–recipient cell‐origin distribution at Day 0, Day 1, Day 3 and Day 5 post‐transplantation. (C) Proportional distribution of major cell populations among donor‐derived, recipient‐derived, and unknown‐origin cells. (D) Proportional analysis of donor‐derived, recipient‐derived, and unknown‐origin cells within each major cell type across different time points. (E) Schematic illustration of BALB/c donor hearts transplanted into CAG‐EGFP C57BL/6 recipient mice, followed by graft collection on Days 3 and 5 for flow cytometry and immunofluorescence analysis. (F) Representative flow cytometry gating strategy for identifying GFP^+^ macrophages among live CD45^+^CD11b^+^F4/80^+^ cells. (G) Representative flow cytometry plots showing GFP^+^ cells among graft‐infiltrating macrophages in negative control, positive control and Day 5 allografts. (H) Quantification of GFP^+^ cells among graft‐infiltrating macrophages. *n* = 3 for negative control, positive controls and Day 5 allografts. (I) Representative immunofluorescence images showing GFP^+^CD68^+^ macrophages in Day 5 allografts. (J) Quantification of GFP^+^CD68^+^ cells among total CD68^+^ cells. *n* = 3 biologically independent samples per group. Data are presented as mean ± SEM. ns, not significant.

To determine the origin of macrophages during cardiac transplantation, we first reanalysed the previously published single‐cell RNA sequencing dataset with donor–recipient cell‐origin annotation. UMAP visualization showed the distribution of donor‐derived, recipient‐derived and unknown‐origin cells in cardiac grafts (Figure 4A). Cell‐origin analysis across different time points further showed that recipient‐derived cells progressively accumulated after transplantation, especially on Days 3 and 5 (Figure 4B). Quantitative analysis of cell composition showed that macrophages accounted for more than 75% of recipient‐derived cell populations (Figure 4C). Further analysis of cell origin within each cell type confirmed that most macrophages in Day 3 and Day 5 grafts were derived from recipients (Figure 4D).

To experimentally validate this finding, BALB/c donor hearts were transplanted into CAG‐EGFP recipient mice, and grafts were collected on Days 5 for flow cytometry and immunofluorescence analysis (Figure 4E). Flow cytometry was performed to identify GFP^+^ macrophages among CD45^+^CD11b^+^F4/80^+^ cells (Figure 4F). Representative plots showed that GFP^+^ cells accounted for 91.7% of graft‐infiltrating macrophages on Day 5 (Figure 4G). Quantitative analysis further confirmed that more than 90% of graft‐infiltrating macrophages were GFP^+^ recipient‐derived cells (Figure 4H). Consistently, immunofluorescence staining showed extensive co‐localization of GFP and CD68 in Day 5 allografts (Figure 4I). Quantification showed that GFP^+^CD68^+^ cells accounted for more than 90% of total CD68^+^ cells (Figure 4J). These findings indicate that the majority of macrophages infiltrating the graft during acute cardiac rejection are derived from the recipient.

### 3.5 Recipient‐derived GSDMD‐deficient macrophages can extend cardiac graft survival

**FIGURE 5:**
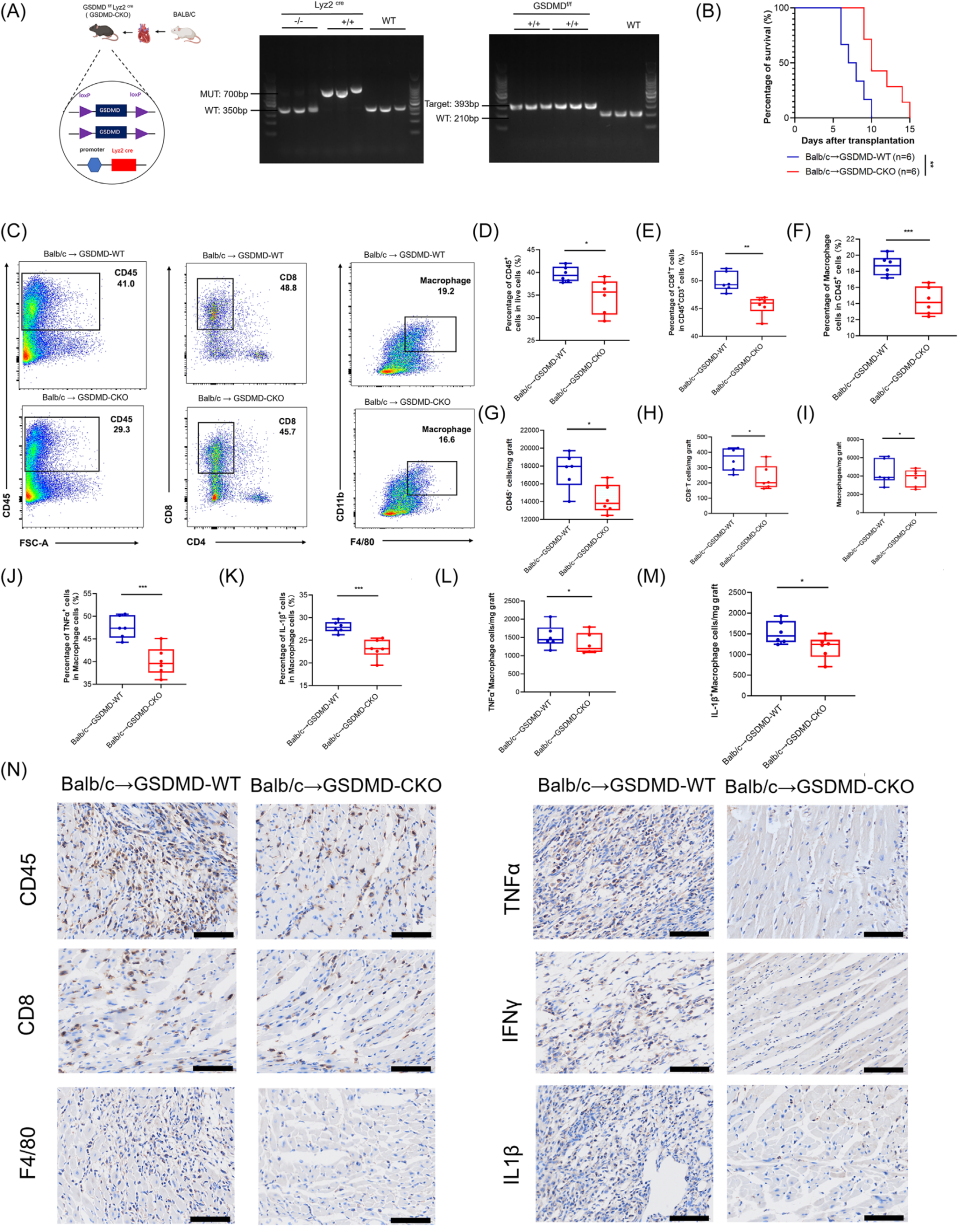
Recipient‐derived GSDMD‐deficient macrophages can prolong cardiac graft survival. (A) Schematic illustration of macrophage‐specific GSDMD knockout mice and PCR‐based genotyping of Lyz2Cre and floxed Gsdmd alleles. For Lyz2Cre genotyping, the mutant allele was detected at 700 bp and the WT allele at 350 bp. For floxed Gsdmd genotyping, the target allele was detected at 393 bp and the WT allele at 210 bp. (B) Survival analysis of cardiac allografts transplanted into GSDMD‐WT or GSDMD‐CKO recipients. *n* = 6 mice per group. (C) Representative flow cytometry plots showing infiltrating CD45^+^ leukocytes, CD8^+^T cells, and macrophages in Day 5 grafts from GSDMD‐WT and GSDMD‐CKO recipients. (D–F) Quantification of the percentages of CD45^+^ leucocytes, CD8^+^T cells, and macrophages in Day 5 grafts. *n* = 6 biologically independent samples per group. (G–I) Quantification of the absolute numbers of CD45^+^ leucocytes, CD8^+^T cells, and macrophages in Day 5 grafts. *n* = 6 biologically independent samples per group. (J and K) Quantification of the percentages of TNF‐α^+^ and IL‐1β^+^ macrophages in Day 5 grafts. *n* = 6 biologically independent samples per group. (L and M) Quantification of the absolute numbers of TNF‐α^+^ and IL‐1β^+^ macrophages in Day 5 grafts. *n* = 6 biologically independent samples per group. (N) Representative immunohistochemical staining of CD45, CD8, F4/80, TNF‐α, IFN‐γ and IL‐1β in Day 5 grafts from GSDMD‐WT and GSDMD‐CKO recipients. Data are presented as mean ± SEM. Survival curves were compared using the log‐rank test. *p < .05; **p < .01; ***p < .001.

To determine whether recipient‐derived macrophage GSDMD contributes to acute cardiac allograft rejection, we generated macrophage‐specific GSDMD‐deficient mice using the Lyz2‐Cre system. PCR genotyping confirmed the successful generation of GSDMD‐CKO mice and corresponding GSDMD‐WT controls (Figure 5A). Before transplantation, we first examined nave GSDMD‐WT and GSDMD‐CKO mice to exclude potential baseline immune differences. Immunohistochemical staining showed no significant differences in CD8^+^T cell or F4/80^+^ macrophage infiltration between nave GSDMD‐WT and GSDMD‐CKO hearts (Figure S5A,B). Flow cytometry analysis of spleens also showed comparable macrophage proportions between the two groups (Figure S5C,D). In addition, the proportions of TNF‐α^+^ and IL‐1β^+^ macrophages were similar between nave GSDMD‐WT and GSDMD‐CKO mice (Figure S5E). Similarly, no significant differences were observed in splenic CD8^+^T cell proportions or in the proportions of TNF‐α^+^ and IFN‐γ^+^ CD8^+^T cells (Figure S5F–H). These findings indicate that macrophage‐specific GSDMD deficiency does not markedly alter baseline immune‐cell composition or cytokine‐producing immune‐cell populations under steady‐state conditions.

We next transplanted BALB/c donor hearts into GSDMD‐WT or GSDMD‐CKO recipients. Compared with GSDMD‐WT recipients, GSDMD‐CKO recipients showed significantly prolonged cardiac allograft survival (Figure 5B). Flow cytometry analysis of Day 5 grafts further showed reduced immune‐cell infiltration in GSDMD‐CKO recipients, with decreased percentages and absolute numbers of CD45^+^ leukocytes, CD8^+^T cells and macrophages in the grafts (Figure 5C–I). Moreover, both the proportions and absolute numbers of TNF‐α^+^ and IL‐1β^+^ macrophages were reduced in GSDMD‐CKO grafts, indicating attenuated macrophage inflammatory activation (Figure 5J–M). Consistently, immunohistochemical staining showed decreased CD45, CD8, F4/80, TNF‐α, IFN‐γ and IL‐1β expression in GSDMD‐CKO grafts compared with GSDMD‐WT grafts (Figure 5N).

We further analysed macrophage polarization in Day 5 grafts. GSDMD‐CKO grafts contained fewer CD86^+^ M1‐like macrophages and more CD206^+^ M2‐like macrophages than GSDMD‐WT grafts, as shown by both proportional and absolute cell number analyses (Figure S6A–E). These data suggest that macrophage‐specific GSDMD deficiency suppresses pro‐inflammatory M1‐like polarization and promotes a shift towards an anti‐inflammatory M2‐like phenotype during acute rejection. Together, these findings indicate that recipient‐derived macrophage GSDMD promotes graft inflammation and rejection, whereas macrophage‐specific GSDMD deficiency prolongs cardiac graft survival by reducing immune‐cell infiltration, inflammatory cytokine production and M1‐like macrophage polarization.

### 3.6 GSDMD‐deficient macrophages attenuated CD8^+^T cell recruitment and activation through IL‐1β

**FIGURE 6:**
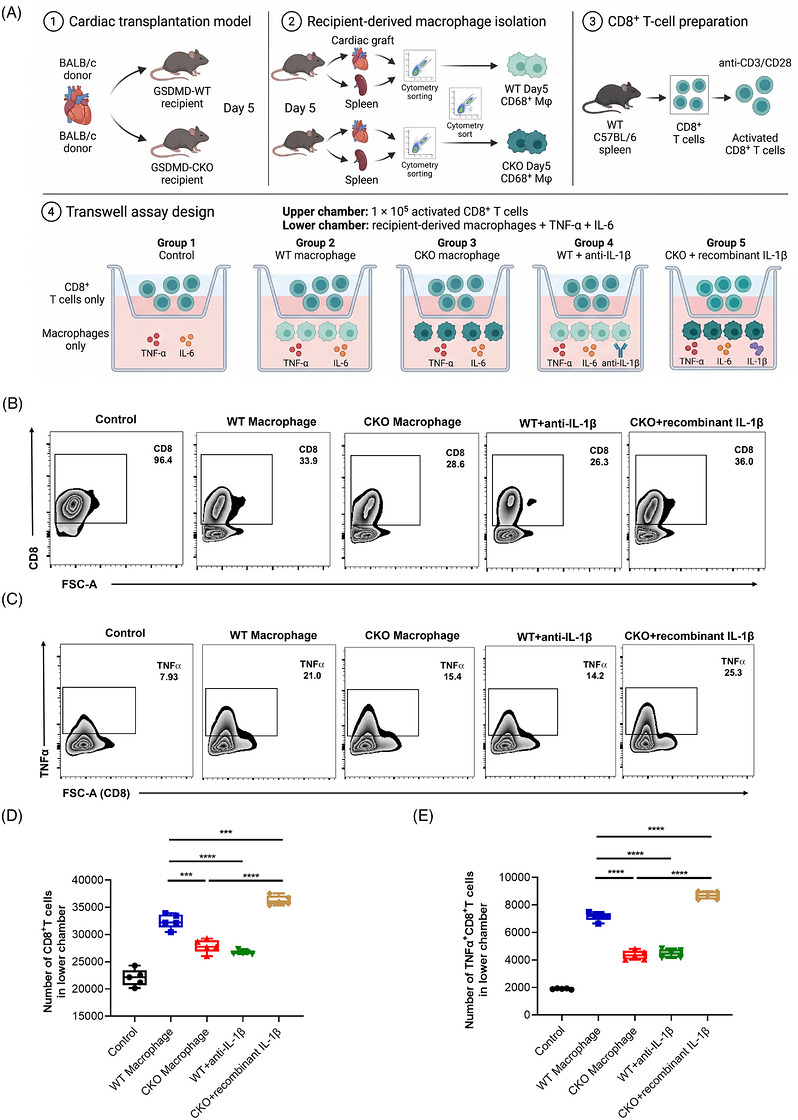
GSDMD‐deficient macrophages attenuated CD8^+^T cell recruitment and activation through IL‐1β. (A) Schematic illustration of the macrophage–CD8^+^T cell Transwell co‐culture assay. BALB/c donor hearts were transplanted into GSDMD‐WT or GSDMD‐CKO recipients. On post‐operative Day 5, CD68^+^ macrophage‐enriched cells were isolated from cardiac grafts and spleens. CD8^+^T cells were isolated from WT C57BL/6 spleens and activated with anti‐CD3/CD28 before co‐culture. Activated CD8^+^T cells were seeded in the upper chamber, and macrophages with TNF‐α and IL‐6 stimulation were placed in the lower chamber. (B) Representative flow cytometry plots showing the percentage of CD8^+^T cells among cells collected from the lower chamber after Transwell co‐culture. (C) Representative flow cytometry plots showing TNF‐α^+^CD8^+^T cells among cells collected from the lower chamber after Transwell co‐culture. (D) Quantification of the number of migrated CD8^+^T cells in the lower chamber. *n* = 6 biologically independent samples per group. (E) Quantification of TNF‐α^+^CD8^+^T cells in the lower chamber. *n* = 6 biologically independent samples per group. Data are presented as mean ± SEM. ***p < .001; ****p < .0001.

To further determine whether recipient‐derived macrophages regulate CD8^+^T cell recruitment and activation during acute rejection, we performed a macrophage–CD8^+^T cell Transwell co‐culture assay. BALB/c donor hearts were transplanted into GSDMD‐WT or GSDMD‐CKO recipient mice, and CD68^+^ macrophage‐enriched cells were isolated from cardiac grafts and spleens on postoperative Day 5. CD8^+^T cells were isolated from WT C57BL/6 spleens and activated with anti‐CD3/CD28 before co‐culture (Figure 6A). Compared with the control group, WT Day 5 macrophages markedly increased the number of migrated CD8^+^T cells in the lower chamber, whereas GSDMD‐CKO macrophages showed a reduced ability to recruit CD8^+^T cells (Figure 6B,D). Neutralization of IL‐1β in the WT macrophage group also decreased CD8^+^T cell migration, while recombinant IL‐1 restored CD8^+^T cell recruitment in the GSDMD‐CKO macrophage group (Figure 6D). Consistently, WT macrophages increased the number of TNF‐α^+^CD8^+^T cells, whereas this effect was attenuated by GSDMD deficiency or IL‐1β neutralization and restored by recombinant IL‐1β supplementation (Figure 6C,E). These findings suggest that recipient‐derived macrophages promote CD8^+^T cell recruitment and functional activation during acute rejection, at least in part through a GSDMD–IL‐1β‐dependent mechanism.

### 3.7 TNF‐α/IL‐6 induces GSDMD upregulation in macrophages via NF‐κB/STAT3 during acute rejection

**FIGURE 7:**
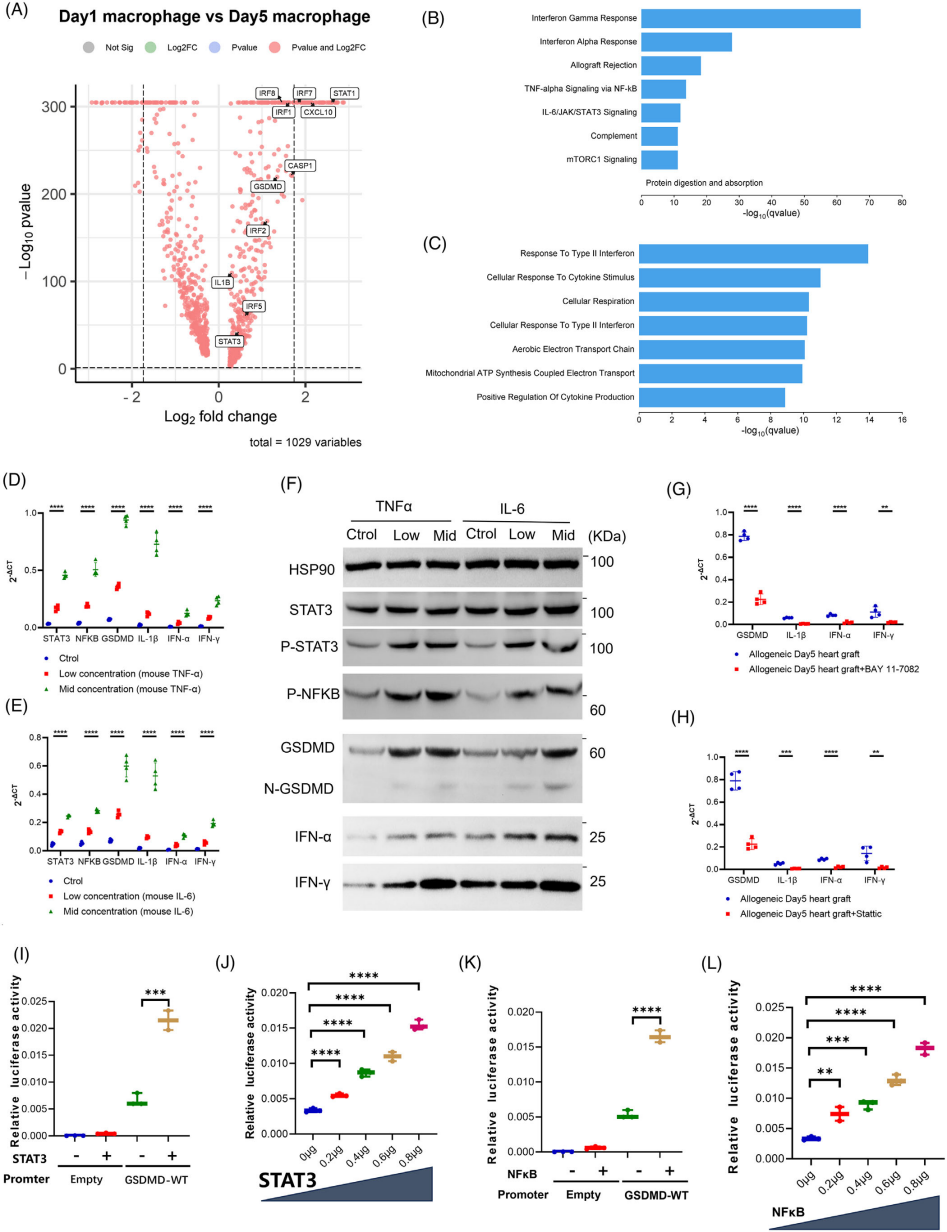
TNF‐α/IL‐6 induces GSDMD upregulation in macrophages via NF‐κB/STAT3 during acute rejection. (A) Volcano plot showing differentially expressed genes in macrophages between Day 1 and Day 5 after transplantation. (B) Pathway enrichment analysis showing activation of inflammatory pathways, including interferon responses, allograft rejection, TNF‐α signalling via NF‐κB, and IL‐6/JAK/STAT3 signalling. (C) Gene ontology analysis showing enrichment of biological processes related to type II interferon response, cellular response to cytokine stimulus, and positive regulation of cytokine production. (D and E) qPCR analysis of Stat3, Nfkb, Gsdmd, Il1b, Ifna and Ifng mRNA expression in RAW264.7 macrophages stimulated with TNF‐α or IL‐6. *n* = 3 independent experiments per group. (F) Western blot analysis of STAT3, p‐STAT3, p‐NF‐κB, GSDMD, N‐GSDMD, IFN‐α and IFN‐γ expression in RAW264.7 macrophages after TNF‐α or IL‐6 stimulation. (G and H) qPCR analysis of Gsdmd, Il1b, Ifna and Ifng mRNA expression in Day 5 allogeneic heart grafts after treatment with BAY 11–7082 or Stattic. *n* = 4 biologically independent samples per group. (I and J) Dual‐luciferase reporter assay showing STAT3‐induced GSDMD promoter activity and dose‐dependent activation of the GSDMD promoter by STAT3. *n* = 3 independent experiments per group. (K and L) Dual‐luciferase reporter assay showing NF‐κB‐induced GSDMD promoter activity and dose‐dependent activation of the GSDMD promoter by NF‐κB. *n* = 3 independent experiments per group. Data are presented as mean ± SEM. **p < .01; ***p < .001; ****p < .0001.

To investigate the molecular mechanism underlying macrophage GSDMD upregulation during acute rejection, we compared the transcriptomic profiles of Day 1 and Day 5 graft‐infiltrating macrophages. Differential expression analysis showed marked upregulation of inflammatory and interferon‐related genes in Day 5 macrophages, including Gsdmd, Il1b, Casp1, Stat1, Stat3, Irf1, and Cxcl10 (Figure 7A). Pathway enrichment analysis revealed activation of inflammatory pathways, including interferon responses (type Ⅱ interferon：IFN‐γ and type Ⅰinterferon：IFN‐α), allograft rejection, TNF‐α/NF‐κB signalling, and IL‐6/JAK/STAT3 signalling (Figure 7B). GO analysis further showed enrichment of cytokine‐ and interferon‐response pathways (Figure 7C), suggesting that inflammatory cytokine signalling may contribute to macrophage GSDMD activation during acute rejection.

To validate this possibility, RAW264.7 macrophages were stimulated with TNF‐α or IL‐6 in vitro. qPCR analysis showed that TNF‐α and IL‐6 increased the expression of Stat3, Nfkb, Gsdmd, Il1b, Ifna and Ifng in a concentration‐dependent manner (Figure 7D,E). Western blot analysis further confirmed that TNF‐α and IL‐6 enhanced STAT3 and NF‐κB activation, as reflected by increased p‐STAT3 and p‐NF‐κB levels, together with increased GSDMD, N‐GSDMD, IFN‐α and IFN‐γ expression (Figure 7F). In vivo, pharmacological inhibition of NF‐κB with BAY 11–7082 or STAT3 with Stattic reduced the expression of Gsdmd, Il1b, Ifna and Ifng in Day 5 allogeneic grafts (Figure 7G,H). Finally, luciferase reporter assays showed that STAT3 and NF‐κB enhanced GSDMD promoter activity, and increasing STAT3 or NF‐κB plasmid concentrations further increased promoter activity in a dose‐dependent manner (Figure 7I–L). These findings suggest that TNF‐α/IL‐6‐associated NF‐κB/STAT3 signalling promotes macrophage GSDMD upregulation during acute cardiac allograft rejection.

### 3.8 IL‐1β can reverse the survival extension of cardiac grafts mediated by macrophage GSDMD deficiency

**FIGURE 8:**
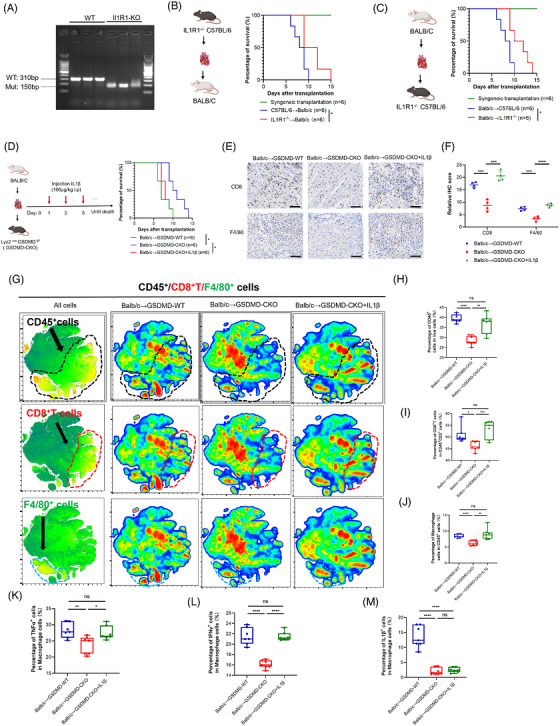
IL‐1β can reverse the survival extension of cardiac grafts mediated by macrophage GSDMD deficiency. (A) PCR‐based genotyping of WT and Il1r1^−/−^ mice. The WT allele was detected at 310 bp, and the mutant allele was detected at 150 bp using allele‐specific PCR. (B) Schematic illustration and survival analysis of WT or Il1r1^−/−^ C57BL/6 donor hearts transplanted into BALB/c recipients. *n* = 6 mice per group. (C) Schematic illustration and survival analysis of BALB/c donor hearts transplanted into WT or Il1r1^−/−^ C57BL/6 recipients. *n* = 6 mice per group. (D) Schematic illustration of recombinant IL‐1β administration in GSDMD‐CKO recipients and survival analysis of cardiac allografts. Recombinant mouse IL‐1β was administered intraperitoneally at 100 µg/kg every other day after transplantation. *n* = 6 mice per group. (E and F) Representative immunohistochemical staining and quantification of CD8 and F4/80 in Day 5 grafts from GSDMD‐WT, GSDMD‐CKO and GSDMD‐CKO + IL‐1β groups. *n* = 4 biologically independent samples per group. (G) Representative flow cytometry density plots showing infiltrating CD45^+^ leukocytes, CD8^+^T cells and F4/80^+^ macrophages in Day 5 grafts from GSDMD‐WT, GSDMD‐CKO and GSDMD‐CKO + IL‐1β groups. (H–J) Quantification of the percentages of CD45^+^ leucocytes, CD8^+^T cells, and macrophages in Day 5 grafts. *n* = 6 biologically independent samples per group. (K–M) Flow cytometric quantification of TNF‐α^+^, IFN‐γ^+^, and IL‐1β^+^ macrophages in Day 5 grafts. *n* = 6 biologically independent samples per group. Data are presented as mean ± SEM. Survival curves were compared using the log‐rank test. ns, not significant; *p < .05; **p < .01; ***p < .001; ****p < .0001.

Given that GSDMD‐mediated macrophage pyroptosis promotes IL‐1β production during cardiac transplantation, we further examined the role of IL‐1β signalling in acute cardiac allograft rejection. PCR genotyping confirmed the successful identification of Il1r1^−/−^ mice (Figure 8A). We first used Il1r1^−/−^ mice as either donors or recipients in cardiac transplantation models. Compared with WT allogeneic transplantation, both Il1r1^−/−^ donor grafts and grafts transplanted into Il1r1^−/−^ recipients showed prolonged survival, suggesting that disruption of IL‐1 signalling attenuates graft rejection (Figure 8B,C).

To determine whether IL‐1β mediates the protective effect of macrophage‐specific GSDMD deficiency, recombinant mouse IL‐1β was administered intraperitoneally to GSDMD‐CKO recipients at 100 µg/kg every other day after transplantation. IL‐1β supplementation shortened graft survival in GSDMD‐CKO recipients, thereby partially reversing the survival benefit conferred by macrophage‐specific GSDMD deficiency (Figure 8D). Immunohistochemical staining showed that GSDMD‐CKO grafts exhibited reduced CD8^+^ T‐cell and F4/80^+^ macrophage infiltration, whereas IL‐1β supplementation restored immune‐cell infiltration in the grafts (Figure 8E,F). Flow cytometry analysis further confirmed that IL‐1β treatment increased the proportions of infiltrating CD45^+^ leucocytes, CD8^+^T cells and macrophages in GSDMD‐CKO grafts (Figure 8G–J).

To address whether IL‐1β supplementation also restored the inflammatory cytokine profile of macrophages, we further analysed cytokine‐positive macrophage populations. GSDMD‐CKO grafts showed reduced proportions of TNF‐α^+^ and IFN‐γ^+^ macrophages compared with GSDMD‐WT grafts, whereas recombinant IL‐1β treatment increased these cytokine‐positive macrophage populations in GSDMD‐CKO grafts (Figure 8K,L). However, the proportion of IL‐1β^+^ macrophages remained low after recombinant IL‐1β treatment (Figure 8 M), suggesting that exogenous IL‐1β mainly restored downstream macrophage inflammatory activation rather than endogenous IL‐1β production. Together, these findings indicate that IL‐1β is an important downstream mediator of macrophage GSDMD‐dependent inflammation and contributes to immune‐cell infiltration and accelerated cardiac allograft rejection.

### 3.9 GSDMD inhibitors can reduce immune cell infiltration and promote graft survival

**FIGURE 9:**
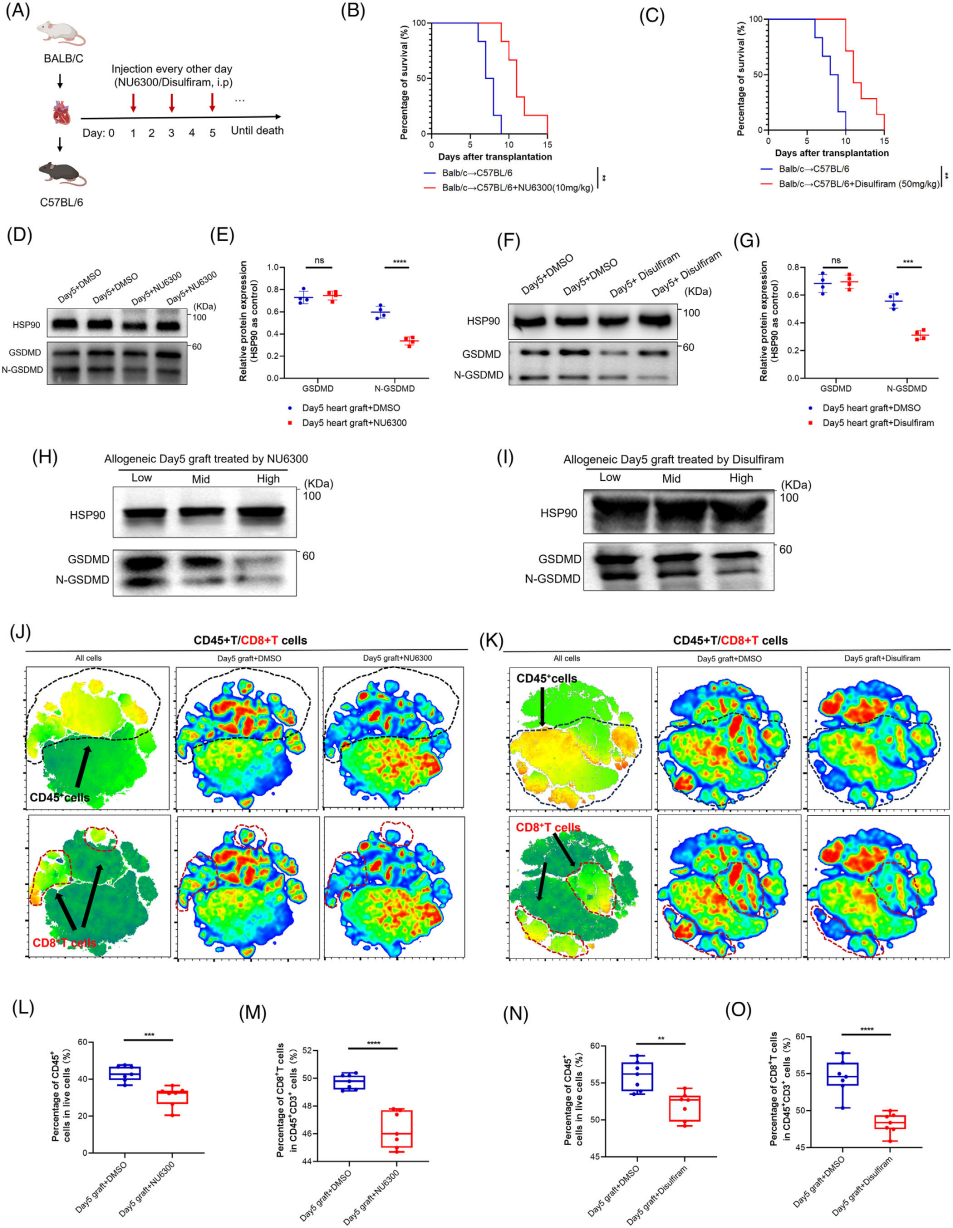
GSDMD inhibitors improved cardiac transplant survival and reduces immune cell infiltration. (A) Schematic illustration of NU6300 or disulfiram administration after cardiac transplantation. NU6300 or disulfiram was administered intraperitoneally every other day after transplantation. (B) Survival analysis of cardiac allografts in vehicle‐ and NU6300‐treated recipients. *n* = 6 mice per group. (C) Survival analysis of cardiac allografts in vehicle‐ and disulfiram‐treated recipients. *n* = 6 mice per group. (D, E) Representative western blot images and quantification of GSDMD and N‐GSDMD expression in Day 5 grafts from vehicle‐ and NU6300‐treated recipients. *n* = 4 biologically independent samples per group. (F, G) Representative western blot images and quantification of GSDMD and N‐GSDMD expression in Day 5 grafts from vehicle‐ and disulfiram‐treated recipients. *n* = 4 biologically independent samples per group. (H) Western blot analysis of GSDMD and N‐GSDMD expression in Day 5 allogeneic grafts treated with increasing doses of NU6300. (I) Western blot analysis of GSDMD and N‐GSDMD expression in Day 5 allogeneic grafts treated with increasing doses of disulfiram. (J and K) Representative flow cytometry density plots showing CD45^+^ leukocytes and CD8^+^T cells in Day 5 grafts after NU6300 or disulfiram treatment. (L and M) Quantification of CD45^+^ leukocytes and CD8^+^T cells in Day 5 grafts from vehicle‐ and NU6300‐treated recipients. *n* = 6 biologically independent samples per group. (N and O) Quantification of CD45^+^ leucocytes and CD8^+^T cells in Day 5 grafts from vehicle‐ and disulfiram‐treated recipients. *n* = 6 biologically independent samples per group. Data are presented as mean ± SEM. Survival curves were compared using the log‐rank test. ns, not significant; **p < .01; ***p < .001; ****p < .0001.

To evaluate whether pharmacological inhibition of GSDMD could attenuate acute cardiac allograft rejection, WT recipients were treated with the GSDMD inhibitors NU6300 or disulfiram after cardiac transplantation. The drug administration schedule is shown in Figure 9A. Compared with the vehicle‐treated group, both NU6300 and disulfiram treatment significantly prolonged cardiac graft survival (Figure 9B,C).

Western blot analysis of Day 5 grafts showed that NU6300 treatment markedly reduced N‐GSDMD expression, while total GSDMD expression was not significantly changed (Figure 9D,E). Similarly, disulfiram treatment decreased N‐GSDMD levels in Day 5 grafts without significantly affecting total GSDMD expression (Figure 9F,G). In addition, dose‐response analysis showed that increasing doses of NU6300 or disulfiram progressively reduced GSDMD cleavage and N‐GSDMD expression in allogeneic Day 5 grafts (Figure 9H,I), supporting effective inhibition of GSDMD‐mediated pyroptosis.

**FIGURE 10:**
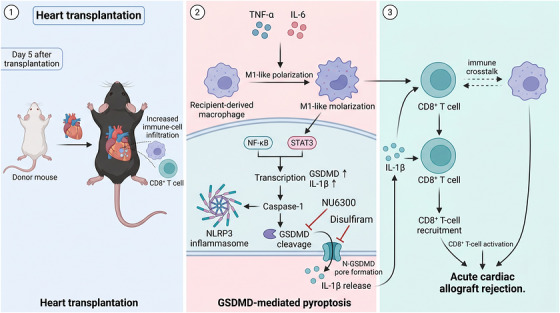
Proposed working model of macrophage GSDMD‐mediated pyroptosis in acute cardiac allograft rejection. After heart transplantation, recipient‐derived macrophages and CD8^+^T cells progressively infiltrate the cardiac graft during the acute rejection stage. Recipient‐derived macrophages in acute rejection undergo M1‐like polarization under the influence of interferon signalling, IL‐6, and TNF‐α. These inflammatory signals activate NF‐κB/STAT3 signalling, leading to increased GSDMD and IL‐1β expression, GSDMD cleavage, N‐GSDMD pore formation, and IL‐1β release. NU6300 and disulfiram inhibit GSDMD‐mediated pyroptosis. Macrophage‐derived IL‐1β further enhances CD8^+^T‐cell recruitment and activation, promoting immune crosstalk between macrophages and T cells and contributing to acute cardiac allograft rejection.

We next examined whether GSDMD inhibition affected graft‐infiltrating immune cells. Flow cytometry analysis showed that NU6300 treatment reduced the percentages of CD45^+^ leucocytes and CD8^+^T cells in Day 5 allografts compared with vehicle treatment (Figure 9J,L,M). Consistently, disulfiram treatment also decreased CD45^+^ leucocyte and CD8^+^T cell infiltration in Day 5 grafts (Figure 9K,N,O). These findings suggest that pharmacological inhibition of GSDMD reduces GSDMD cleavage, attenuates immune‐cell infiltration, and prolongs cardiac allograft survival during acute rejection. Together, these findings support a working model in which recipient‐derived macrophage GSDMD‐mediated pyroptosis promotes IL‐1β release, CD8^+^ T cell activation and acute cardiac allograft rejection (Figure 10).

## 4 DISCUSSION

Our previous study described the landscape of pyroptosis in cardiac allografts during acute cellular rejection. The analysis revealed that GSDMD‐mediated pyroptosis was significantly elevated during the acute rejection phase. Based on this finding, we further explored the immunological role of GSDMD‐mediated pyroptosis in cardiac transplantation. Single‐cell RNA sequencing (scRNA‐seq) analysis showed that macrophages underwent a shift from the M2 to M1 phenotype during acute rejection, with a significant increase in GSDMD expression in M1 macrophages. Therefore, targeting GSDMD in macrophages could be a potential therapeutic strategy to extend graft survival. The underlying mechanism may involve the chemotaxis of macrophages from the recipient's circulation to the graft, where they are activated by inflammatory factors (such as TNF‐α and IL‐6) that upregulate GSDMD expression, providing the necessary proteins for pyroptosis activation. Pyroptosis in macrophages promotes the release of inflammatory cytokine IL‐1β, further exacerbating the levels of other inflammatory mediators and triggering an inflammatory storm. Targeting GSDMD can effectively block this process, reducing immune cell infiltration and inflammatory cytokine levels, thus extending graft survival. In a word, the process begins with the activation of pro‐inflammatory pathways by cytokines such as IL‐1β, TNFα and IFN‐α/β/γ, which induce M1 polarization of recipient‐derived macrophages. This polarization leads to increased GSDMD‐mediated pyroptosis, culminating in the promotion of immune rejection through enhanced IL‐1β production, further amplifying inflammation and graft rejection.

Pyroptosis is a form of programmed cell death mediated by the classical pyroptotic proteins GSDMD and GSDME, often associated with the release of numerous inflammatory cytokines.^29^ The level of pyroptosis can be assessed by the expression of the activated fragments N‐GSDMD/N‐GSDME, which are produced upon cleavage of GSDMD/GSDME.^30^One recent study proved that GSDME‐mediated pyroptosis of macrophage accelerates the progression of atherosclerosis and its associated inflammation.^31^ In this study, we primarily focussed on GSDMD‐mediated pyroptosis of macrophage. Our findings showed that during ischemia‐reperfusion, the levels of pyroptosis remained relatively stable, whereas GSDMD‐mediated pyroptosis significantly increased during acute rejection. Further experiments indicated that this increase in pyroptosis was closely linked to the elevation of inflammatory cytokines such as TNF‐α and IL‐6 during the acute rejection phase, which activate the NF‐κB/STAT3‐GSDMD pathway. To investigate whether targeting GSDMD could improve graft survival, we used GSDMD^−/−^ mice as recipients. We observed that grafts from GSDMD^−/−^ recipients showed a substantial decrease in GSDMD‐mediated pyroptosis. This was accompanied by a significant reduction in immune cell infiltration (such as CD8^+^T cells and macrophages) and a decrease in pro‐inflammatory cytokines like TNF‐α and IL‐1β, which led to a marked extension in graft survival. These findings suggest that the pyroptosis observed in the graft during acute rejection originates from recipient‐derived cells that migrate into the graft. To further investigate this mechanism, we performed single‐cell RNA sequencing (scRNA‐seq) to analyse macrophage populations at Days 0, 1, 3 and 5 post‐transplantation. The scRNA‐seq results showed a temporal shift in macrophage phenotypes, with resident macrophages predominating on Day 0, while M1 macrophages gradually increased by Days 1 and 3. By Day 5, during acute rejection, most macrophages exhibited a classic pro‐inflammatory M1 phenotype. We also observed that GSDMD expression was elevated specifically in M1 macrophages at Day 5. Immunofluorescence staining confirmed this observation. Using GFP‐KI mice as recipients, we found that 90% of the macrophages during both ischemia–reperfusion and acute rejection phases originated from the recipient and migrated into the graft. Previous studies have shown that inflammatory cytokines such as TNF‐α, IL‐1β, IL‐6 and IFN‐α/β/γ are the key factors in driving the M2‐to‐M1 conversion of macrophages. Based on our findings, we conclude that during acute rejection on Day 5, the increase in these cytokines promotes the conversion of macrophages from M2 to M1, with the M1 macrophages migrating into the graft. This process is accompanied by increased GSDMD expression, leading to amplified GSDMD‐mediated pyroptosis.

In our subsequent experiments, we used GSDMD‐CKO mice as recipients in cardiac transplantation studies. We found that GSDMD deficiency in macrophages significantly prolonged graft survival. Flow cytometry and immunohistochemistry revealed reduced infiltration of CD8^+^T cells and macrophages, along with diminished secretion of pro‐inflammatory cytokines TNF‐α and IL‐1β by macrophages, thus reducing the severity of acute rejection. Single‐cell RNA sequencing of macrophages on Day 5 revealed activation of the TNFα–NF‐κB and IL6‐STAT3 signalling pathways, which likely contribute to the upregulation of GSDMD expression. Luciferase reporter assays confirmed that both NF‐κB and STAT3 activation can drive GSDMD transcription. Since IL‐1β is a key inflammatory cytokine involved in GSDMD‐mediated pyroptosis, we further investigated the role of IL‐1β in graft survival using IL‐1R1^−/−^ mice in the transplant model. Our results showed that IL‐1R1^−/−^ recipients, whether as donors or recipients, experienced significantly prolonged graft survival. In addition, we performed macrophage–CD8^+^T cell chemotaxis and co‐culture assays, which showed that macrophage‐derived inflammatory signals, particularly IL‐1β, promoted CD8^+^T cell recruitment and functional activation. These findings support a crosstalk model in which GSDMD‐mediated macrophage pyroptosis contributes to downstream CD8^+^T cell responses during acute cardiac allograft rejection. In contrast, the administration of recombinant IL‐1β to GSDMD‐CKO recipients reversed the prolonged graft survival, indicating that IL‐1β is a critical mediator of the extended graft survival observed in GSDMD‐CKO mice.

GSDMD inhibitors, including disulfiram and NU6300, act through two primary mechanisms: one is direct binding to GSDMD to prevent its activation, and the other is inhibition of the N‐terminal fragment to prevent membrane pore formation.^32^ Previous studies have shown that both disulfiram and NU6300 specifically bind to the Cys191 site of GSDMD, forming covalent bonds that prevent Caspase‐1‐mediated GSDMD cleavage, thereby reducing the release of inflammatory cytokines such as IL‐1β and IL‐18.^33^, ^34^ These compounds have demonstrated anti‐inflammatory effects in models of inflammatory bowel disease (IBD) and other chronic inflammatory conditions by inhibiting GSDMD‐mediated pyroptosis.^35^ In an acute lung injury model, disulfiram reduced tissue damage by targeting GSDMD‐dependent pyroptosis.^36^ In our experiment, treatment with disulfiram and NU6300 in cardiac transplant models resulted in extended graft survival by significantly decreasing N‐GSDMD‐mediated pyroptosis and IL‐1β infiltration, highlighting the therapeutic potential of GSDMD inhibition in improving cardiac transplant outcomes.

We acknowledge several limitations in this study. First, our research was conducted using a mouse cardiac transplantation model, and the lack of human cardiac transplant samples presents challenges for further validation. Second, while we focussed on GSDMD, the role of GSDME remains underexplored and warrants further investigation. Third, IL1R1 knockout mice lack response to both IL‐1α and IL‐1β, so the contribution of IL‐1α cannot be excluded. In addition, the use of only male mice as recipients may introduce sex‐based differences in T cell immune responses, potentially influencing the results. Finally, one limitation of this study is that disulfiram and NU6300 may have potential off‐target effects in vivo. Thus, the inhibitor experiments should be interpreted as supportive pharmacological evidence rather than definitive proof of GSDMD specificity. Nevertheless, the findings from global and macrophage‐specific GSDMD‐deficient mice, together with IL‐1β rescue and macrophage–CD8^+^T cell co‐culture assays, support the role of GSDMD‐mediated macrophage pyroptosis in acute cardiac allograft rejection. Future studies using more selective GSDMD inhibitors or macrophage‐targeted delivery strategies are needed to further validate the therapeutic specificity of GSDMD inhibition. Despite these limitations, our study highlights the novel role of cardiac macrophages in cardiac inflammation and transplant immunity, offering valuable insights into the immunological mechanisms of cardiac transplantation. Future studies should explore whether GSDMD inhibitors combined with established immunosuppressive regimens can further improve graft survival and enhance the translational potential of targeting GSDMD‐mediated pyroptosis.

In conclusion, our research sheds light on the function of cardiac macrophages in transplantation and the impact of GSDMD‐mediated pyroptosis. These findings enhance our understanding of immune responses in heart and other organ transplants, providing new theoretical and therapeutic avenues for improving transplant outcomes.

## AUTHOR CONTRIBUTIONS

**Bixian Luo**: Writing—original draft preparation. **Bixian luo, Zelai Wu, Chengyu Hu** and **Anqi Ni**: Writing—review and editing. **Weixun Xie** and **Jun He**: visualization. **Hongming Liu**: Supervision. **Weihua Gong**: Administration. **Weihua Gong**: Funding acquisition. All authors have read and agreed to the published version of the manuscript.

## CONFLICT OF INTEREST STATEMENT

The authors declare no conflicts of interest.

## FUNDING INFORMATION

This study was supported by the Key Research and Development Program of Zhejiang Province (grant no.: 2025C02054) and the National Natural Science Foundation of China (grant no.: 82470416).

## ETHICS STATEMENT

All procedures involving pathological information usage and relative patient informed consent record were according to the guideline of Ethics Review Board of second affiliated hospital of school of medicine, Zhejiang university. All animal experiments were approved by the Zhejiang University Animal Center and conducted under the protocol license (IACUC‐ZJU20220256).

## Supporting information



Supporting Information

Supporting Information

## Data Availability

The original contributions presented in the study are included in the article/supplementary materials, further inquiries can be directed to the corresponding author.
