# Supplementary material for: Recipient‐derived macrophages mediate acute cardiac allograft rejection via GSDMD‐induced pyroptosis mechanism
Source: Clin Transl Med. 2026 Jul 5;16(7):e70729. doi: 10.1002/ctm2.70729 (PMC13334137; doi:10.1002/ctm2.70729)
Supplement: Supplementary file 1 — Supporting Information [file CTM2-16-e70729-s002.docx]

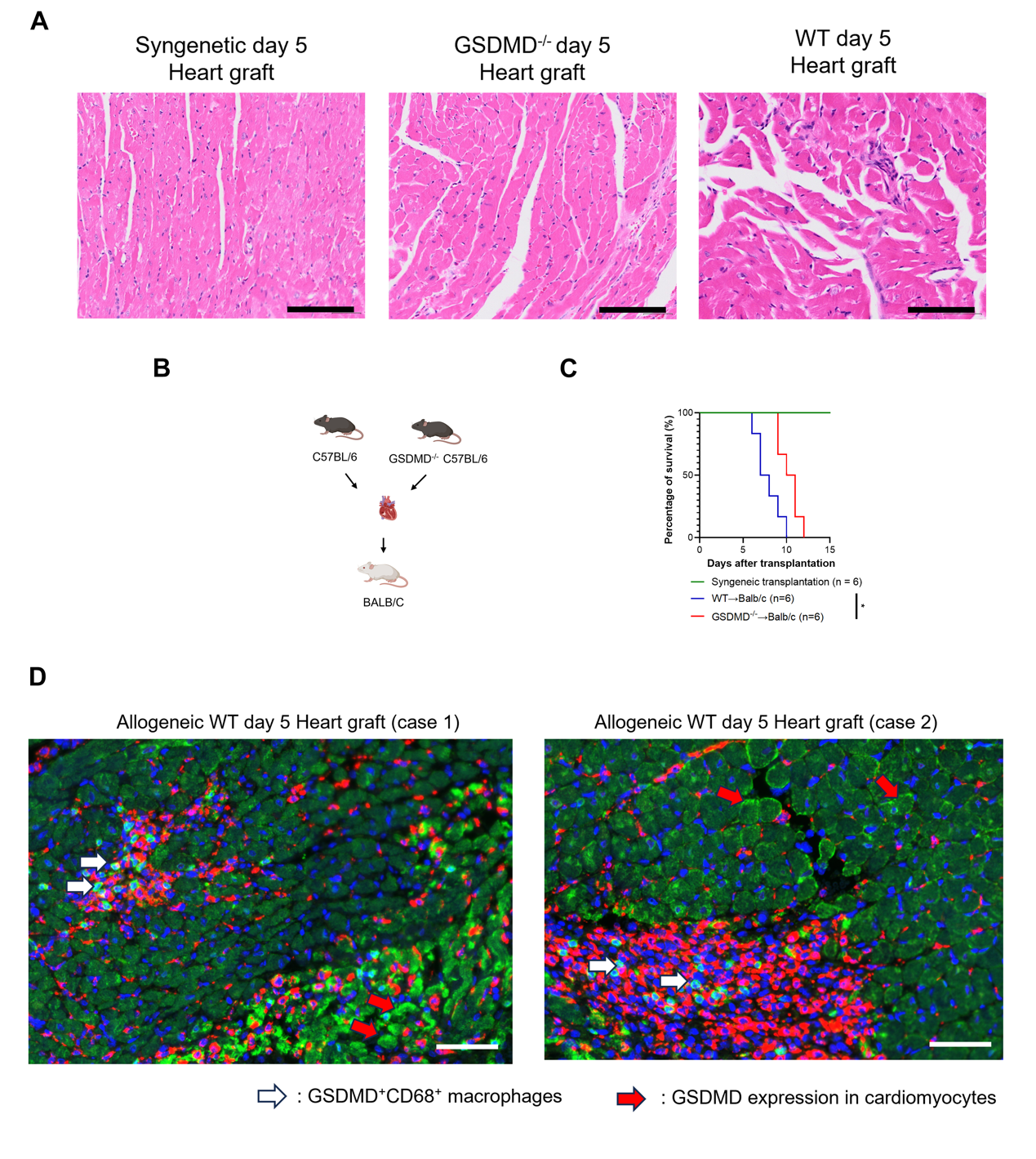


**Supplementary Figure 1. Histological assessment, donor GSDMD deficiency, and GSDMD localization in cardiac allografts.**
(A) Representative H&E staining of syngeneic Day 5 grafts, allogeneic Gsdmd^-/-^ Day 5 grafts, and allogeneic WT Day 5 grafts. (B) Schematic illustration of WT or Gsdmd^-/-^ C57BL/6 donor hearts transplanted into BALB/c recipients. (C) Survival analysis showing prolonged graft survival of Gsdmd^-/-^ donor hearts compared with WT donor hearts. n = 6 mice per group. (D) Representative immunofluorescence images showing GSDMD⁺CD68⁺ macrophages and GSDMD expression in cardiomyocytes in Day 5 allogeneic WT grafts. Images from two representative biological cases are shown. White arrows indicate GSDMD⁺CD68⁺ macrophages, and red arrows indicate GSDMD expression in cardiomyocytes. ns, not significant; *P < 0.05.

**Supplementary Figure 2. Single-cell annotation and temporal changes in graft cell populations.**
(A) Dot plot showing the average expression levels of canonical marker genes used for major cell-type annotation, including macrophages (Lyz2, Cd68, Aif1, Cd163, and Fcgr1), cardiac fibroblasts (Col3a1, Col1a1, and Pdgfra), pericytes (Pdgfrb and Kcnj8), T cells (Cd3e and Cd3g), granulocytes (S100a9 and Ccr1), B cells (Ms4a1, Cd19, and Cd79a), endothelial cells (Pecam1 and Cdh5), and smooth muscle cells (Acta2, Myh11, and Tagln). (B) UMAP visualization of major cell populations in cardiac grafts. (C) UMAP visualization of graft cells colored by sampling time points. (D) Proportional distribution of major cell populations across Day 0, Day 1, Day 3, and Day 5 post-transplantation. (E) Feature plots showing the expression of macrophage marker genes Cd68 and Lyz2 in graft cells.


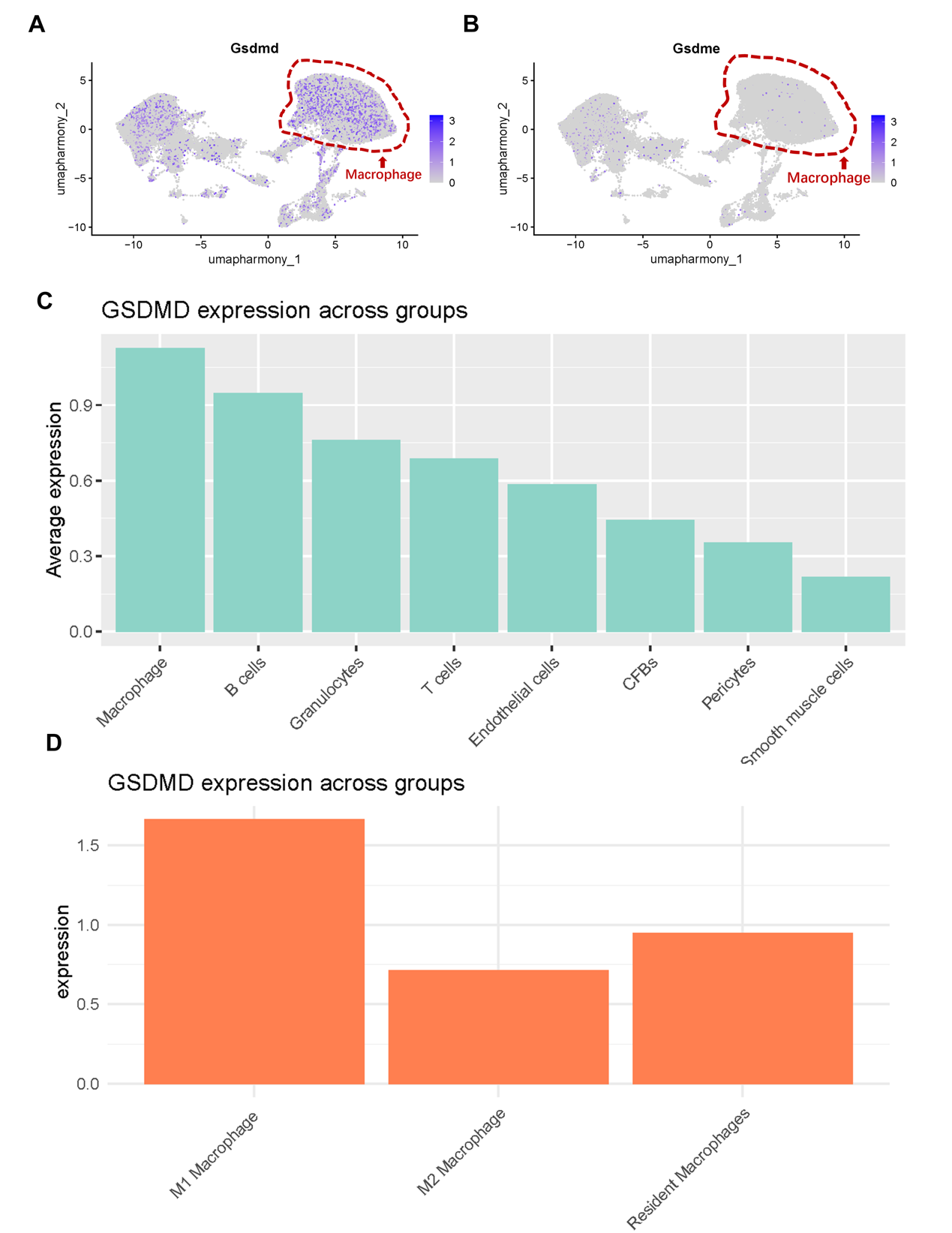


**Supplementary Figure 3. Gsdmd expression was enriched in macrophages and M1-like macrophage subsets.**
(A, B) Feature plots showing Gsdmd and Gsdme expression in graft cells. Macrophage-enriched regions are indicated by dashed red outlines. (C) Average Gsdmd expression across major graft cell populations. (D) Average Gsdmd expression across M1-like, M2-like, and resident macrophage populations.


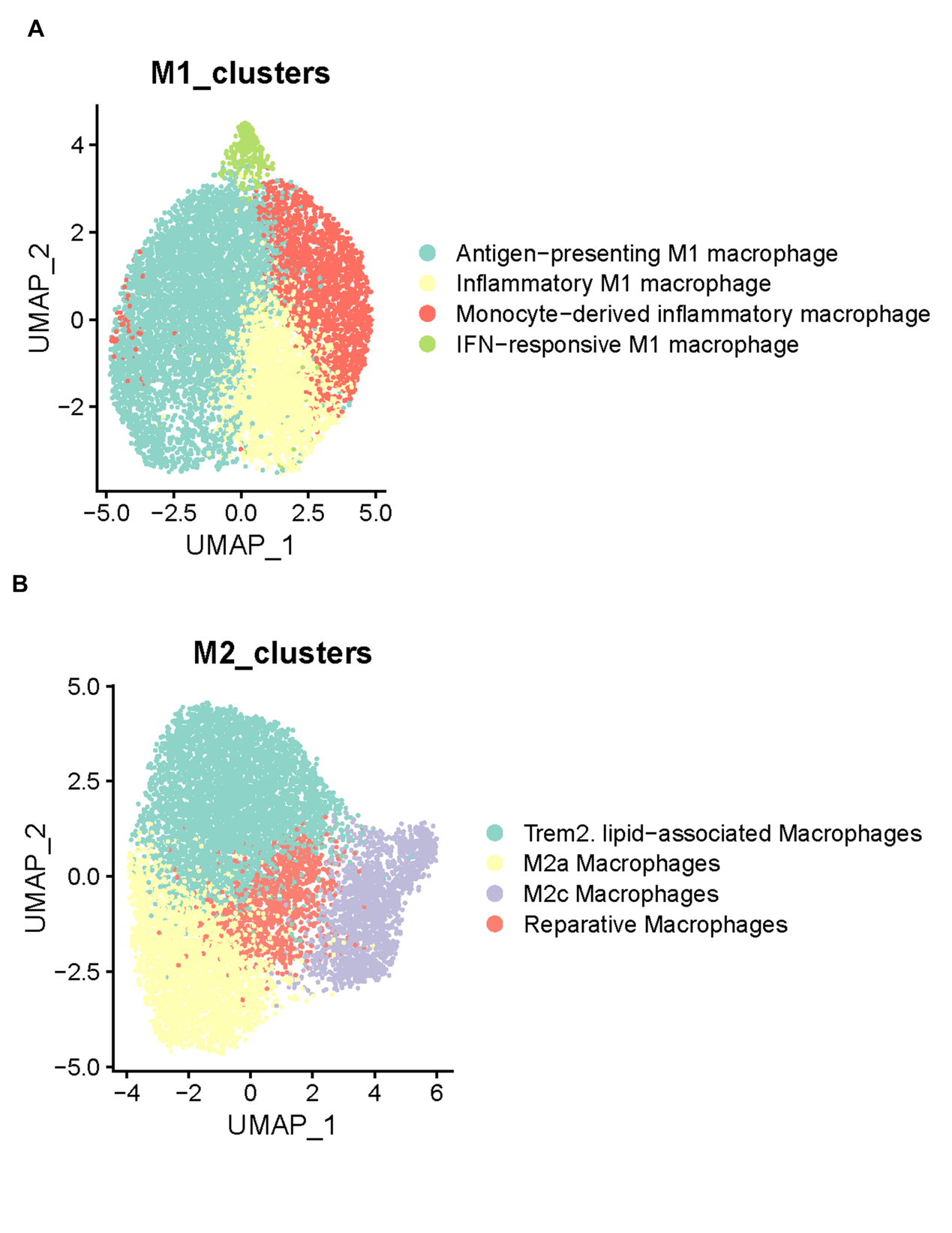


**Supplementary Figure 4. Subclustering of M1-like and M2-like macrophage populations.**
(A) UMAP visualization of M1-like macrophage subclusters, including antigen-presenting M1-like macrophages, inflammatory M1-like macrophages, monocyte-derived inflammatory macrophages, and IFN-responsive M1-like macrophages. (B) UMAP visualization of M2-like macrophage subclusters, including Trem2-associated lipid-associated macrophages, M2a macrophages, M2c macrophages, and reparative macrophages.


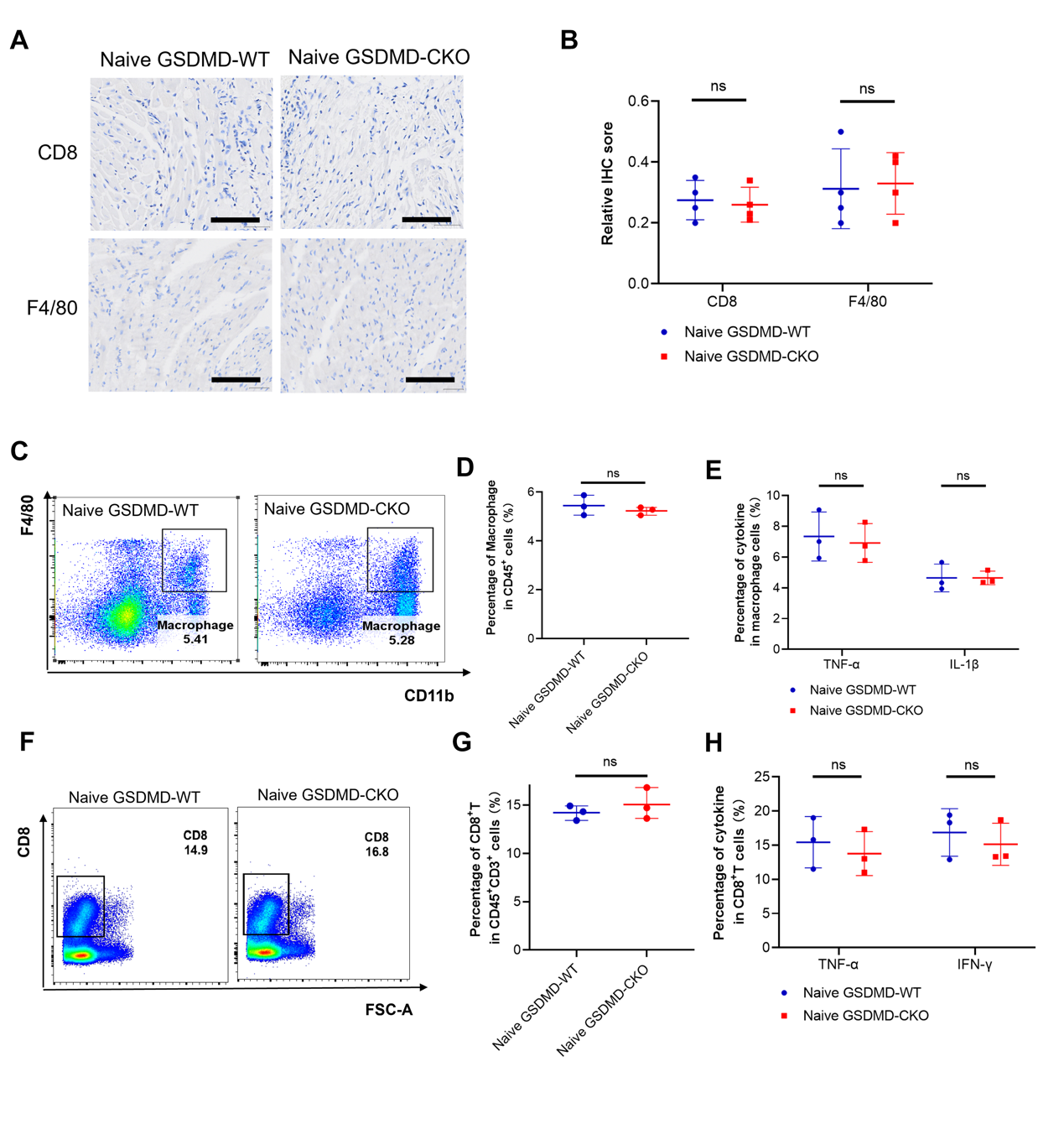


**Supplementary Figure 5. Baseline immune-cell profiles were comparable between naïve GSDMD-WT and GSDMD-CKO mice.**
(A, B) Representative immunohistochemical staining and quantification of CD8 and F4/80 in naïve GSDMD-WT and GSDMD-CKO hearts. n = 4 biologically independent samples per group. (C, D) Representative flow cytometry plots and quantification of macrophages in naïve GSDMD-WT and GSDMD-CKO spleens. n = 3 biologically independent samples per group. (E) Flow cytometric quantification of TNF-α⁺ and IL-1β⁺ macrophages in naïve GSDMD-WT and GSDMD-CKO spleens. n = 3 biologically independent samples per group. (F, G) Representative flow cytometry plots and quantification of CD8⁺ T cells in naïve GSDMD-WT and GSDMD-CKO spleens. n = 3 biologically independent samples per group. (H) Flow cytometric quantification of TNF-α⁺ and IFN-γ⁺ CD8⁺ T cells in naïve GSDMD-WT and GSDMD-CKO spleens. n = 3 biologically independent samples per group. Data are presented as mean ± SEM. ns, not significant.


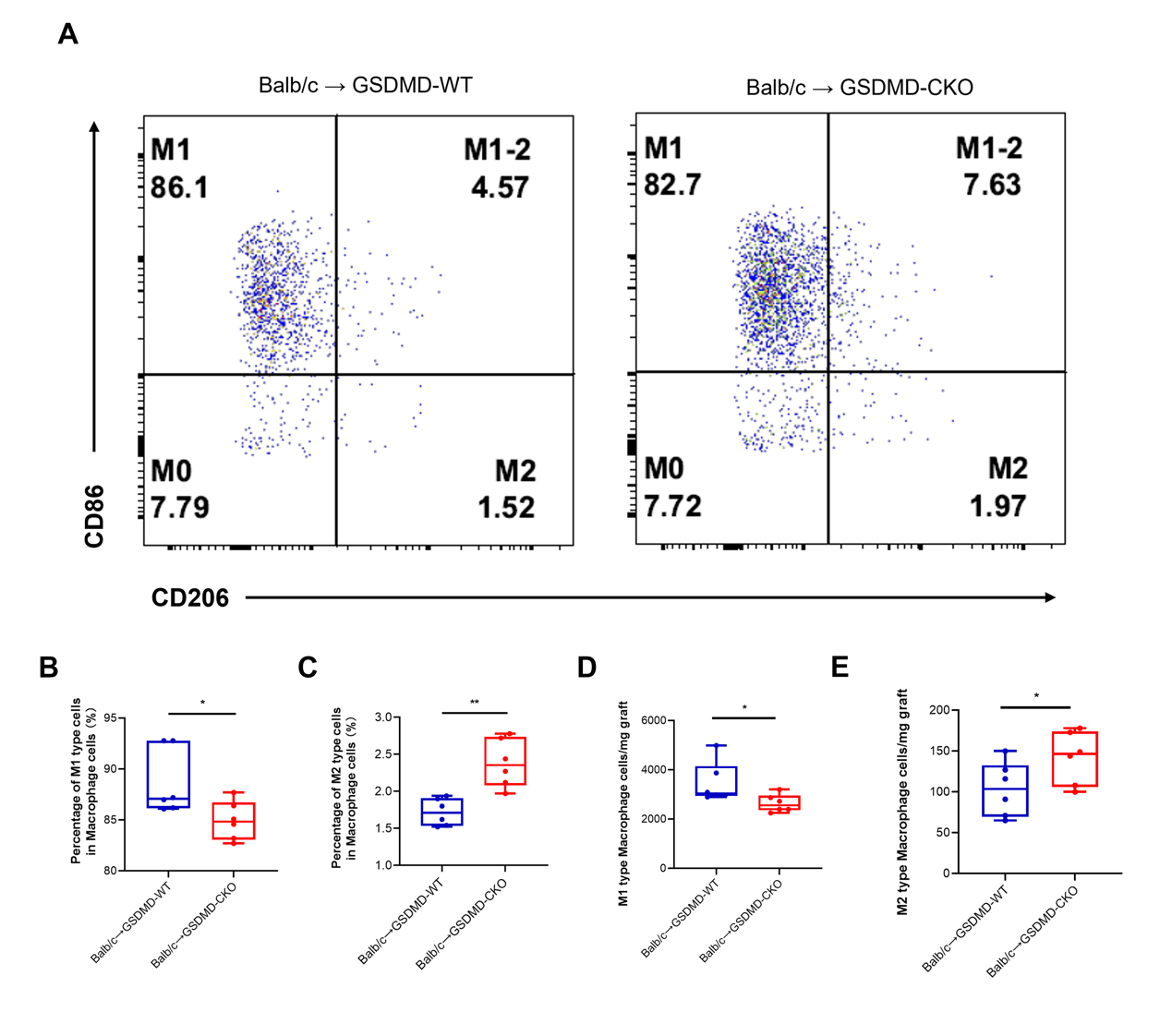
**Supplementary Figure 6. Macrophage-specific GSDMD deficiency attenuated M1-like macrophage polarization in cardiac allografts.**
(A) Representative flow cytometry plots showing CD86 and CD206 expression in graft-infiltrating macrophages from GSDMD-WT and GSDMD-CKO recipients. (B, C) Quantification of the percentages of CD86⁺ M1-like and CD206⁺ M2-like macrophages among graft-infiltrating macrophages. (D, E) Quantification of the absolute numbers of CD86⁺ M1-like and CD206⁺ M2-like macrophages in Day 5 grafts. n = 5 biologically independent samples per group. Data are presented as mean ± SEM. *P < 0.05; **P < 0.01.
