# Supplementary material for: Recipient‐derived macrophages mediate acute cardiac allograft rejection via GSDMD‐induced pyroptosis mechanism
Source: Clin Transl Med. 2026 Jul 5;16(7):e70729. doi: 10.1002/ctm2.70729 (PMC13334137; doi:10.1002/ctm2.70729)
Supplement: Supplementary file 2 — Supporting Information [file CTM2-16-e70729-s001.docx]

| **Supplementary table 1: Antibodies used in western blot.** | | |  |
| --- | --- | --- | --- |
| Antibodies | Source | Identifier | |
| Anti-GSDMD | affinity | AF4012 | |
| Anti-N-GSDMD | affinity | DF13758 | |
| Anti-HSP90 | Cell signaling technology | #4877 | |
| Anti-HSP90 | Abcam | ab203126 | |
| Anti-GSDMD | Abcam | ab209845 | |
| Anti-N-GSDMD | Abcam | ab215203 | |
| Anti-STAT3 | Cell signaling technology | #9319 | |
| Anti-P-STAT3 | Cell signaling technology | #9145 | |
| Anti-P- NF-κB | Cell signaling technology | #3033 | |
| Anti-P- NF-κB | Cell signaling technology | #4806 | |
| Anti- IFNα | Abcam | ab232899 | |
| Anti-IFNβ | Cell signaling technology | #97450 | |
| Anti- IFNγ | Cell signaling technology | #98139 | |
| Anti-ACTIN | Cell signaling technology | #4970s | |
| Anti-IL-1B | Abcam | ab234437 | |
| Anti-IL18 | Abcam | ab207323 | |

**Supplementary table 2. Antibodies used for flow cytometry.**

| **Reagent / Antibody** | **Fluorochrome** | **Source** | **Catalog No.** |
| --- | --- | --- | --- |
| LIVE/DEAD Zombie Aqua™ Fixable Viability Kit | Zombie Aqua | BioLegend, USA | 423101 |
| anti-mouse CD45 | BV605 | BioLegend, USA | 103139 |
| anti-mouse CD45 | BV421 | BioLegend, USA | 103133 |
| anti-mouse CD45 | APC-Cy7 | BioLegend, USA | 103115 |
| anti-mouse CD3 | APC-Cy7 | BioLegend, USA | 100221 |
| anti-mouse CD3 | BV421 | BioLegend, USA | 100227 |
| anti-mouse CD8a | BV421 | BioLegend, USA | 100737 |
| anti-mouse CD8a | PE-Cy7 | BioLegend, USA | 100721 |
| anti-mouse CD4 | PE-Cy7 | BioLegend, USA | 100527 |
| anti-mouse CD4 | BV786 | BD Biosciences, USA | 563727 |
| anti-mouse CD11b | BV785 | BioLegend, USA | 101243 |
| anti-mouse CD11b | FITC | BioLegend, USA | 101205 |
| anti-mouse F4/80 | RB613 | BD Biosciences, USA | 759375 |
| anti-mouse F4/80 | BV421 | BioLegend, USA | 123131 |
| anti-mouse CD86 | PE | BioLegend, USA | 105007 |
| anti-mouse CD86 | APC | BioLegend, USA | 105011 |
| anti-mouse CD206/MMR | PE | BioLegend, USA | 141705 |
| anti-mouse CD206/MMR | APC | BioLegend, USA | 141707 |
| anti-mouse IL-1β pro-form | PE | Thermo Fisher Scientific/eBioscience, USA | 12-7114-82 |
| anti-mouse IL-1β pro-form | APC | Thermo Fisher Scientific/eBioscience, USA | 17-7114-80 |
| anti-mouse TNF-α | APC | BioLegend, USA | 506307 |
| anti-mouse TNF-α | PE | BioLegend, USA | 506305 |
| anti-mouse IFN-γ | Spark UV™ 387 | BioLegend, USA | 505863 |
| anti-mouse IFN-γ | PerCP-Cy5.5 | BioLegend, USA | 505821 |

**Supplementary table 3: Primer sequences of genes for qPCR.**

| Genes | Direction | Base sequence |
| --- | --- | --- |
| m-GSDMD | Forward | CCATCGGCCTTTGAGAAAGTG |
|  | Reverse | ACACATGAATAACGGGGTTTCC |
| m-STAT3 | Forward | AGCTGGACACACGCTACCT |
|  | Reverse | AGGAATCGGCTATATTGCTGGT |
| m-NF-κB | Forward | CCATCGGCCTTTGAGAAAGTG |
|  | Reverse | ACACATGAATAACGGGGTTTCC |
| m-GAPDH | Forward | AGGTCGGTGTGAACGGATTTG |
|  | Reverse | TGTAGACCATGTAGTTGAGGTCA |
| β-ACTIN | Forward | CTGGAACGGTGAAGGTGACA |
|  | Reverse | AAGGGACTTCCTGTAACAATGCA |
| m-IFNβ | Forward | CAGCTCCAAGAAAGGACGAAC |
|  | Reverse | GGCAGTGTAACTCTTCTGCAT |
| m-IFNγ | Forward | ATGAACGCTACACACTGCATC |
|  | Reverse | CCATCCTTTTGCCAGTTCCTC |
| m-IFNα | Forward | TGATGAGCTACTACTGGTCAGC |
|  | Reverse | GATCTCTTAGCACAAGGATGGC |
| m-IL1β | Forward | GCAACTGTTCCTGAACTCAACT |
|  | Reverse | ATCTTTTGGGGTCCGTCAACT |
| m-IL6 | Forward | CCAAGAGGTGAGTGCTTCCC |
|  | Reverse | CTGTTGTTCAGACTCTCTCCCT |
| m-IL18 | Forward | GACTCTTGCGTCAACTTCAAGG |
|  | Reverse | CAGGCTGTCTTTTGTCAACGA |
| m-TNFα | Forward | GACGTGGAACTGGCAGAAGAG |
|  | Reverse | TTGGTGGTTTGTGAGTGTGAG |

| **Supplementary table 4: antibodies for IHC and IFC analysis** | | |
| --- | --- | --- |
| Antibodies | Source | Identifier |
| Anti-CD8 | Servicebio | GB15068 |
| Anti-CD45 | Servicebio | GB14038 |
| Anti-F4/80 | Servicebio | GB11027 |
| Anti-CD68 | Servicebio | GB113109 |
| Anti-TNFα | Servicebio | GB115701 |
| Anti-IL1B | Servicebio | GB112059 |
| Anti- IFNγ | affinity | DF6045 |
| Anti-GSDMD | Abcam | ab209845 |
| Anti-N-GSDMD | Abcam | ab215203 |
| Anti-GSDMD | affinity | AF4012 |
| Anti-N-GSDMD | affinity | DF13758 |
